# Genomic analysis of multidrug-resistant *Escherichia coli* from Urban Environmental water sources in Accra, Ghana, Provides Insights into public health implications

**DOI:** 10.1371/journal.pone.0301531

**Published:** 2024-05-24

**Authors:** Rebecca Tettey, Beverly Egyir, Prudence Tettey, John Arko-Mensah, Samuel Ofori Addo, Christian Owusu-Nyantakyi, William Boateng, Julius Fobil

**Affiliations:** 1 Department of Biological, Environmental, and Occupational Health Science, School of Public Health, College of Health Sciences, University of Ghana, Accra, Ghana; 2 West African Center for Global Environmental & Occupational Health, College of Health Sciences, University of Ghana, Accra, Ghana; 3 Department of Bacteriology, Noguchi Memorial Institute for Medical Research, College of Health Sciences, University of Ghana, Accra, Ghana; North Carolina State University, UNITED STATES

## Abstract

Wastewater discharge into the environment in resource-poor countries poses a threat to public health. Studies in this area within these countries are limited, and the use of high-throughput whole-genome sequencing technologies is lacking. Therefore, understanding of environmental impacts is inadequate. The present study investigated the antibiotic resistance profiles and diversity of beta-lactamases in *Escherichia coli* strains isolated from environmental water sources in Accra, Ghana. Microbiological analyses were conducted on wastewater samples from three hospitals, a sewage and wastewater treatment plant, and water samples from two urban surface water bodies. Confirmed isolates (N = 57) were selected for phenotypic antibiotic resistance profiles. Multi-drug-resistant isolates (n = 25) were genome sequenced using Illumina MiSeq sequencing technology and screened for sequence types, antibiotic resistance, virulence and beta-lactamase genes, and mobile genetic elements. Isolates were frequently resistant to ampicillin (63%), meropenem (47%), azithromycin (46%), and sulfamethoxazole-trimethoprim (42%). Twenty different sequence types (STs) were identified, including clinically relevant ones such as ST167 and ST21. Five isolates were assigned to novel STs: ST14531 (n = 2), ST14536, ST14537, and ST14538. The isolates belonged to phylogroups A (52%), B1 (44%), and B2 (4%) and carried β-lactamase (TEM-1B, TEM-1C, CTX-M-15, and blaDHA-1) and carbapenemase (OXA-1, OXA-181) resistance genes. Dominant plasmid replicons included Col440I (10.2%) and IncFIB (AP001918) (6.8%). Polluted urban environments in Accra are reservoirs for antibiotic-resistant bacteria, posing a substantial public health risk. The findings underscore the need for targeted public health interventions to mitigate the spread of antibiotic-resistant bacteria and protect public health.

## Introduction

Antibiotic resistance (AR) poses a threat human and animal health, casting uncertainty on the attainment of sustainable development goals– 1, 2, 3, 6, 10, 14, and 15–[1]. Whereas AR was previously believed to be associated with the misuse of antibiotics in healthcare and animal husbandry, the current pervasiveness of antibiotics (parent compounds and functional metabolites) in the natural environment [2,3] implies that other sources are involved [4,5]. There are reports that the increased levels of AR in urban environments in low- and middle-income countries (LMICs) may be due to the lack of proper waste management systems, a major concern in the fight against antimicrobial resistance (AMR) [6,7]. Additionally, untreated or partially treated waste containing antibiotic-resistant bacteria (ABR) and antibiotic-resistance genes (ARGs) is discharged into the environment together with other micropollutants, including heavy or toxic metals from the use of personal care products and other anthropogenic activities. The discharge of these chemical compounds and biological agents transforms the environment into hotspots for the acquisition, transfer, and dissemination of ARGs between pathogenic and environmental bacteria via horizontal gene transfer (HGT) [5,8]. These facts emphasize the need to investigate potential sources of ARB and emerging ARGs of clinical relevance circulating in environments where they could easily spread to humans and animals directly or indirectly [9].

Aquatic ecosystems are recognized as hotspots for the development and dissemination of AR [10,11]. These environments may pose a health risk because of their proximity or the benefits derived by nearby communities from them. Antibiotic-resistant bacteria can spread from these environments to recreational waters, drinking water, and farm produce through rainwater run-off and flooding. For example, multidrug-resistant (MDR) pathogenic *E*. *coli* causing human and animal infections have been documented in the environment in Ghana and elsewhere [6,12–14]. However, studies utilizing sequencing technologies to study environmental *E*. *coli* or other bacteria are limited in Africa [14]. Ghana, in particular, currently lacks these data because studies employing whole genome sequencing (WGS) technologies to characterise ARGs in environmental bacteria are rare. Most studies typically report phenotypic AMR data and polymerase chain reaction (PCR) analyses [12,15,16]. Consequently, these studies may be limited in their scope of ARGs investigated and the complexity of their relationships [17]. As a result, there is insufficient information about potential or emerging pathogens and ARGs from the environment that could spread to clinically important pathogens, leading to infections that are difficult to treat. The World Health Organization (WHO) has compiled a list of antibiotic-resistant "priority pathogens" with the aim of facilitating and advancing research and development of new antibiotics. This list specifically emphasizes the significant threat posed by Gram-negative bacteria exhibiting MDR. The most critical group includes clinically important MDR bacteria belonging to the Enterobacteriaceae family, such as *E*. *coli* [18,19].

*Escherichia coli* is often used as an indicator of fecal contamination of water [20] because it is a non-pathogenic bacterium that colonizes the gastrointestinal tract of humans and other animals. It becomes pathogenic by acquiring specific virulence factors [21]. Pathotypes causing intestinal or extraintestinal infections in humans pose a public health risk. A biphasic lifestyle, with host-associated and host-independent phases, is an adaptive feature that enables *E*. *coli* to survive and contribute to the spread of ARGs in natural environments [22]. Furthermore, there are reports of its high resistance level to older human and veterinary antibiotics, such as tetracycline, ampicillin, and streptomycin, and an increasing level of resistance to newer antibiotics, such as cephalosporins and fluoroquinolones [23]. Multidrug-resistant *E*. *coli* isolates are those that are resistant to at least one agent in three or more antibiotic classes [24]. *Escherichia coli* can become resistant to antibiotics through chromosomal mutations or by acquiring resistance factors such as mobile genetic elements (MGEs) from closely related or unrelated bacterial species. For instance, the tetracycline resistance gene, *tet(M)*, primarily found on transposons in Gram-positive bacteria, was also observed on plasmids in *E*. *coli* from a natural river basin [25]. Additionally, the most common Enterobacteriaceae β-lactamases, *blaSHV*, *blaTEM*, and *blaCTX-M*, are also common with *E*. *coli* isolates [26].

The aim of this study was to comprehensively analyze the genomics of *E*. *coli* strains isolated from polluted environmental sources to assess the potential risks posed to public health and safety. In this study, we examined AR patterns, phylogenic diversity, VAGs, and their association with MGEs of *E*. *coli* in wastewater samples collected from hospitals and a wastewater treatment plant (WWTP) that discharges into one of the water bodies included in this study, as well as surface water from two water bodies. Additionally, previous reports [12,27] have shown that environmental water sources heavily impacted by human activities have the potential to harbor and transmit ARB. Hence, we hypothesize that *E*. *coli* isolated from environmental sources will show a greater prevalence of resistance to the antibiotics compared with those isolated from a less impacted environmental water source.

## Materials and methods

### Ethical approval

Ethical approval was not required for this study because only environmental samples were used; no human participants or animal models were involved.

### Study site description

Samples were collected from the Odaw River (OD) and the Korle Lagoon (KL), effluents from a sewage treatment plant, and three hospitals in Accra, Ghana’s capital city. Additionally, samples were collected from a pristine site known as the Wli waterfalls, which is situated approximately 189.2 km away from Accra, to serve as a reference site. The waterfall serves as a conservation and tourism attraction site and is less impacted by intense human activity compared to the other sampling sites.

The hospitals comprise a primary and two tertiary healthcare facilities with bed capacities of 2000, 400, and 130, respectively, in Accra. The OD, a major river with other tributaries, originates in the Eastern Region of Ghana and flows through Accra. It is marked by intense human activity along its course: industries, agricultural areas (vegetable farms and animal ranches), transport terminals with shopping centers, open markets, and health facilities. Along its course are slums known for open defaecation and indiscriminate disposal of refuse, urban areas, and health centers. Agbogbloshie, popularly known for informal electronic waste recycling, is a major landmark in its catchment area. Located a short distance from the riverbanks lies a previously abandoned landfill site that has undergone reclamation efforts and has been transformed into a thriving vegetable garden by the urban underprivileged community. This community uses water sourced from the river to irrigate the cultivated vegetables.

The OD flows into the KL, ultimately draining into the Gulf of Guinea. A sewage and wastewater treatment plant (WWTP) and a drainage system, gathering water from nearby communities within the KL catchment area, discharge midway along the KL stream. These two water bodies were previously significant providers of fish and opportunities for recreational activities. However, they have undergone severe pollution and are presently unsuitable for human use. They now sustain only a limited number of fish species, namely *Clarias gariepinus* and *Oreochromis niloticus*. These water bodies could serve as hotspots for the development and spread of ARB in these settings and beyond. Therefore, it is imperative to conduct studies that evaluate the impact of waste on the dissemination of ARB and ARG within these ecosystems.

### Study design and sample collection

A two-time study was undertaken in Accra, Ghana during the dry (January) and wet (July) seasons of 2021. A total of 150 samples comprising wastewater samples (n = 12) collected at the points of discharge from the three hospitals, and from a municipal sewage and WWTP (n = 8) at the point of discharge into the KL.

The selection of hospitals was guided by the understanding that hospital wastewater serves as a significant reservoir for MDR pathogens, subsequently discharged directly into drains that ultimately feed into the two water bodies. Different levels of health facilities were chosen to showcase the varying tiers of healthcare available, all of which discharge into the aforementioned water bodies.

Manual grab water and sediments samples were collected from the WWTP and hospital waste water because they discharge into the OD and the KL, which are two main water bodies that flow through Accra Central. Sampling from the WWTP and hospital effluents (HE) aimed at source tracking to determine the occurrence of the target organisms before their release into the receiving water bodies. Sampling was conducted across multiple points within the same source to obtain representative samples.

Surface water and sediment samples were collected because reports [28,29] indicate that during regular flow conditions, approximately 20% to 35% of indicator microorganisms in the aquatic environment are linked to suspended particles that have the potential to settle. During storm events, these values experience an increase ranging from 30 to 55%. Hence, failure to consider sediment quality when monitoring surface water quality in the aquatic ecosystem may result in an underestimation of the true microbial load at sampling sites. This underestimation could pose a potential human health risk when sediment resuspension occurs, releasing bacteria into the surface water [30].

**Fig 1:**
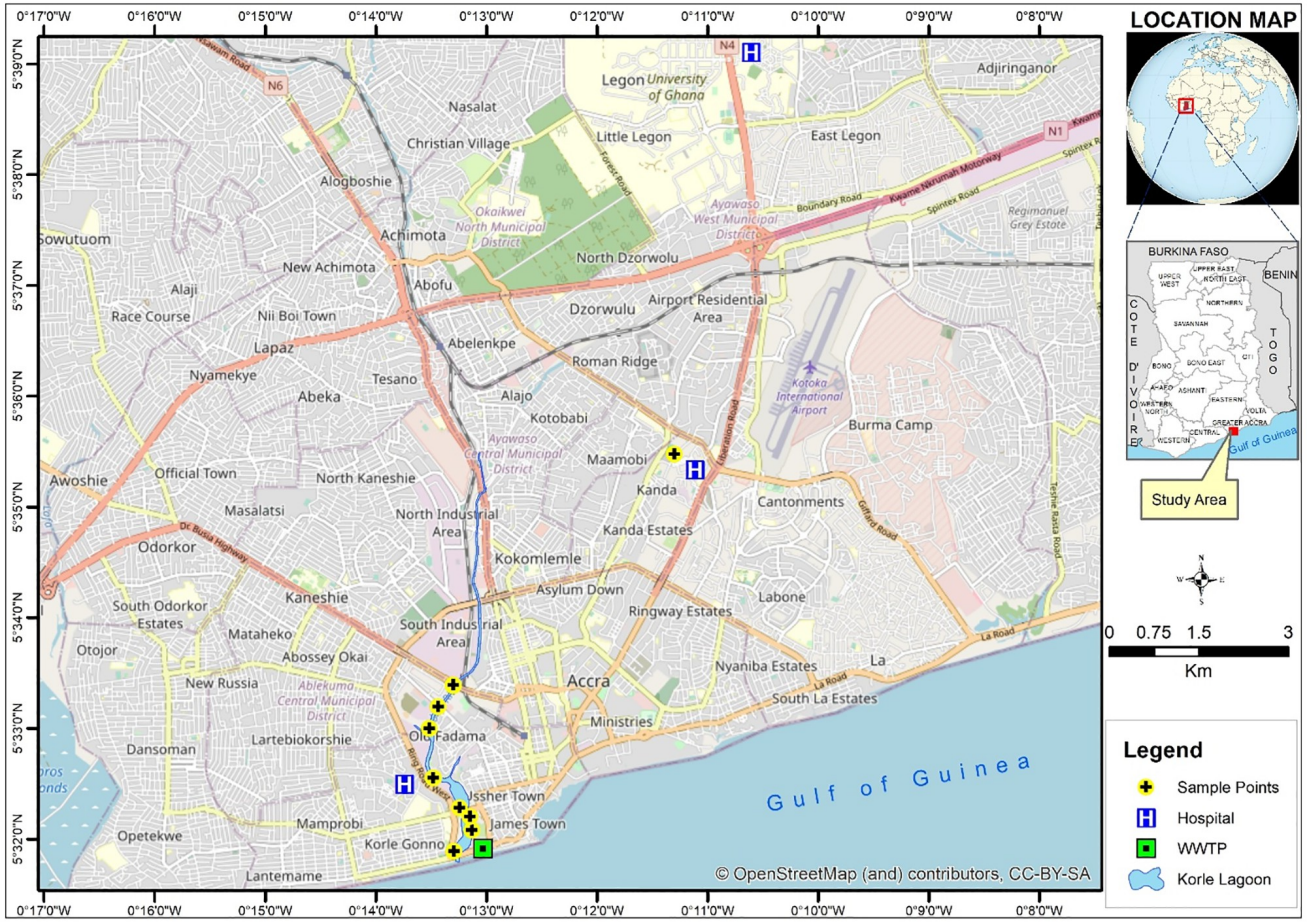
Map of Accra showing the sampling sites. Yellow markings indicate a few of the sampling sites.

Surface water (n = 18) and sediment (n = 18) samples were also collected from the OD and the KL (S1 Table; Fig 1). Water and sediment samples were collected from the Wli waterfalls (n = 18). Water samples for microbiological analysis were collected with a stainless-steel bucket into 500-mL sterile borosilicate bottles and capped [31]. Sediment samples (about 50g each) were collected alongside the water samples by pushing down a hollow cylindrical tube to a depth of 0–10 cm. Sediment samples were homogenized and transferred into sterile sample bags (Whirl-Pak^®^, Merck, Germany). Samples were appropriately labelled and documented in a field notebook as well as a sampling form before leaving the sampling areas.

Samples were placed were chilled at 4°C in a portable icebox and transported to the laboratory. They were stored at 4°C prior to analysis and transported to the laboratory within six hours after collection in order to maintain their temperature at 4°C.

### Isolation and identification of *Escherichia coli*

Standard microbiological methods were used for *E*. *coli* isolation and identification. Water samples were mixed thoroughly and serially diluted by transferring 1 ml of the sample to 9 ml of 0.9% sterile saline, followed by subsequent serial dilutions (10^−1^ to 10^−6^). Chromogenic coliform agar (CCA; Oxoid Limited, UK) plates were inoculated with 0.1 ml each of 10^−5^ and 10^−6^ dilutions, followed by surface spreading and incubation at 37°C for 24 h. Additionally, 10g of sediment was transferred to 90 ml of sterile saline, mixed vigorously by vortexing for 30 s, and allowed to settle. The supernatant was serially diluted, followed by inoculation and incubation of CCA plates as described above. Characteristic blue/purple colonies indicative of *E*. *coli* colonies were Gram stained and Gram-negative colonies were further confirmed by subculturing on Eosin Methylene Blue Agar (EMBA, Oxoid Limited, UK) at 37°C for 24 h. The growth of colonies with a green metallic sheen was presumptive of *E*. *coli*. Secondary identification was performed using pure *E*. *coli* colonies using matrix-assisted laser desorption/ionization-time of flight mass spectrometry (MALDITOF MS, Bruker Daltonik GmbH, Leipzig, Germany). Briefly, the surface of the colony to be identified was touched with a sterile loop to make a smear on the steel target and air dried for 15 min. The resulting smears were overlaid with 1 μl of 70% formic acid and air dried. Subsequently, they were overlaid again with 1μl of matrix solution (containing cyano-4- hydroxy-cinnamic acid in 50% acetonitrile with 2.5% trifluoroacetic acid) and air dried for 15 min. The steel target plate was placed in the high-vacuum source chamber of the Microflex LT benchtop instrument, which was operated using FlexControl software (Bruker Daltonik GmbH, Leipzig, Germany). As an internal control, a 0.5 L Bacterial Test Standard (Bruker Daltonics Inc Billerica, MA) was used. The results were saved and printed. Protein identities were assigned using a CSU-PMF internal library, where a 70% match of total mass-spectral peaks (score of >2.0) identified an isolate as *E*. *coli* [32]. Isolates confirmed as *E*. *coli* were subjected to further downstream analyses.

### Antibiotic susceptibility testing

The antibiotic susceptibility testing (AST) of the identified *E*. *coli* isolates was conducted with a panel of 12 antibiotic discs (Oxoid, UK), including chloramphenicol (C30), ampicillin (AMP10), ciprofloxacin (CIP5), trimethoprim-sulfamethodazole (STX_1.25_/_23.75_), cefotaxime (CTX30), ceftazidime (CAZ30), ceftriaxone (CRO30), cefuroxime (CXM30), meropenem (MEM10), fosfomycin (FOF200), azithromycin (AZM15), and amikacin (AK30). These antibiotics were chosen based on the Clinical and Laboratory Standards Institute (CLSI) guidelines for antibiotic susceptibility testing (AST) of Enterobacterales [33] and because they have been frequently used in the treatment of infections caused by Gram-negative bacteria in human and veterinary medicine in Ghana. The disc diffusion method was employed. To increase the reliability of the results, *E*. *coli* ATCC 25922 was used as a positive control. The CLSI breakpoint charts were followed for the interpretation of minimum inhibitory concentrations (MICs) and zone diameters [33]. Colonies showing resistance to three or more antibiotic discs, considered MDR, were selected and prepared for sequencing [24].

### Confirmation of extended spectrum beta Lactamase-*E*. *coli*

*Escherichia coli* isolates that exhibited resistance to multiple cephalosporins were suspected of being Extended Spectrum Beta Lactamase (ESBL) producers [zone diameter of ≤27 mm for cefotaxime, ≤22 mm for ceftazidime, and ≤25 mm for ceftriaxone around the discs]. In accordance with the CLSI guidelines [33], the double-disc synergy test (DDST) was employed to validate the ESBL production of suspected colonies. Combination discs containing cefotaxime (30 μg) and ceftazidime (30 μg)/clavulanic acid (10 μg) were positioned on the seeded MH plates, approximately 24 mm away from their corresponding single discs (ceftazidime and cefotaxime, each at 30 μg). The MH plates were subsequently incubated for 16–18 hours at 37°C. To substantiate the test results, the *E*. *coli* ATCC 25922 reference strain, which lacked ESBL activity, was incubated concurrently with the experiment. Extended Spectrum Beta Lactamase production was deemed to have occurred when the diameter of the combination discs increased by more than 5 mm in comparison to their individual single discs.

### Genomic DNA extraction and whole genome sequencing

Of the 43 *E*. *coli* isolates obtained, 25 (58%) were MDR and were selected for WGS comprised six (24%), four (16%), 12 (48%), and three (12%) were selected from HE, KL, OD, and WWTP, respectively. DNA extraction and WGS were performed following methods described previously [34]. Briefly, DNA was extracted using the QIAamp DNA Mini Kit (Qiagen Inc., GmbH, Holden, Germany) and quantified using a Qubit TM 4.0 Fluorometer (Invitrogen, Thermo Fischer Scientific, Singapore). The library was prepared using the Nextera DNA flex library preparation kit (Illumina, San Diego, CA, USA). An Agilent 2100 bioanalyzer system (Agilent, Santa Clara, CA, USA) and quantitative PCR (qPCR) using the Kapa Sybr Fast qPCR kit (Kapa Biosystems, USA) were used to measure the quality and concentration of fragmented DNA amplified libraries, respectively. Libraries were pooled and loaded on the Illumina 2 x 300 cycle for sequencing on the Mi-Seq machine (Illumina Inc., San Diego, CA, USA).

### Bioinformatic analyses

The raw sequenced reads were subjected to quality filtering and trimming using FASTQC (https://www.bioinformatics.babraham.ac.uk/projects/fastqc/) and Trimmomatic V0.39 [35]. The minimum quality threshold was set at Q20 [36]. Trimmed reads underwent de novo assembly using the Unicycler V0.4.8 assembler with default parameters. Only contigs with a length greater than 200 bp were retained for subsequent analysis.

All contiguous sequences were subsequently submitted to GenBank and assigned accession numbers under BioProject PRJNA900446. The assembled genomes were analysed for multilocus sequence typing (MLST) sequence types (STs) on the MLST 2.0 database hosted by the Center for Genomic Epidemiology (CGE) (http://cge.cbs.dtu.dk/services/MLST/) using default parameters. Isolates without STs were proffered to the EnteroBase Escherichia/Shigella database (https://enterobase.warwick.ac.uk/species/index/ecoli) and assigned novel STs. The acquired resistance gene types or chromosomal mutation mediating resistance were analysed using ResFinder 4.1 hosted by CGE (https://cge.food.dtu.dk/services/ResFinder/) using default parameters. Insertion sequences were determined using ISFinder. FimH and FumC types were assigned using CHTyper 1.0 (https://cge.food.dtu.dk/services/CHTyper/) using default parameters. The phylogroups of the isolates were determined using the ClermonTyping database (http://clermontyping.iame-research.center/) using default parameters.

### Phylogenetic analyses

Whole genome sequences of isolates were uploaded and analysed using REALPHY 1.13, hosted by the Swiss Institute of Bioinformatics and the Universität Basel Center for Molecular Life Sciences (https://realphy.unibas.ch/realphy/). *Salmonella enterica subsp*. *enterica serovar Typhimurium str*. The LT2 genome (Accession number: PRJNA57799) was used as an outgroup to root the tree, allowing the phylogenetic distance between isolates and branches to be easily configured. To provide insights into the generated tree, the phylogeny was visualized and annotated based on the metadata using iTOL V6 (https://itol.embl.de/tree/). Furthermore, a phylogenetic analysis generated on the basis of single-nucleotide polymorphisms (SNPs) of the core genes of *E*. *coli* isolates from this study and 48 isolates (S2A and S2B Tables) comprising those from human, poultry, and pigs from previous studies in Ghana, curated at the BB-BRC 3.30.19a website (https://www.bv-brc.org/view/Taxonomy/561#view_tab=genomes) was performed to understand the clonal relationship between these isolates as well as the epidemiological significance of isolates from this study.

## Results

### Distribution of *Escherichia coli* isolates across all studied sources

A total of 150 water and sediment samples were cultured, resulting in 57 *E*. *coli* isolates [61.4% (35/57) from OD, 18% (10/57) from KL, 9% (5/57) from WWTP, and 12.3% (7/57) from HE] *Escherichia coli* was not isolated from samples from the pristine site; instead, the isolates were observed to be *Citrobacter freundii* and were not included in further downstream analysis.

### Antibiotic susceptibility profiles and characterization of the ESBLs

**Fig 2:**
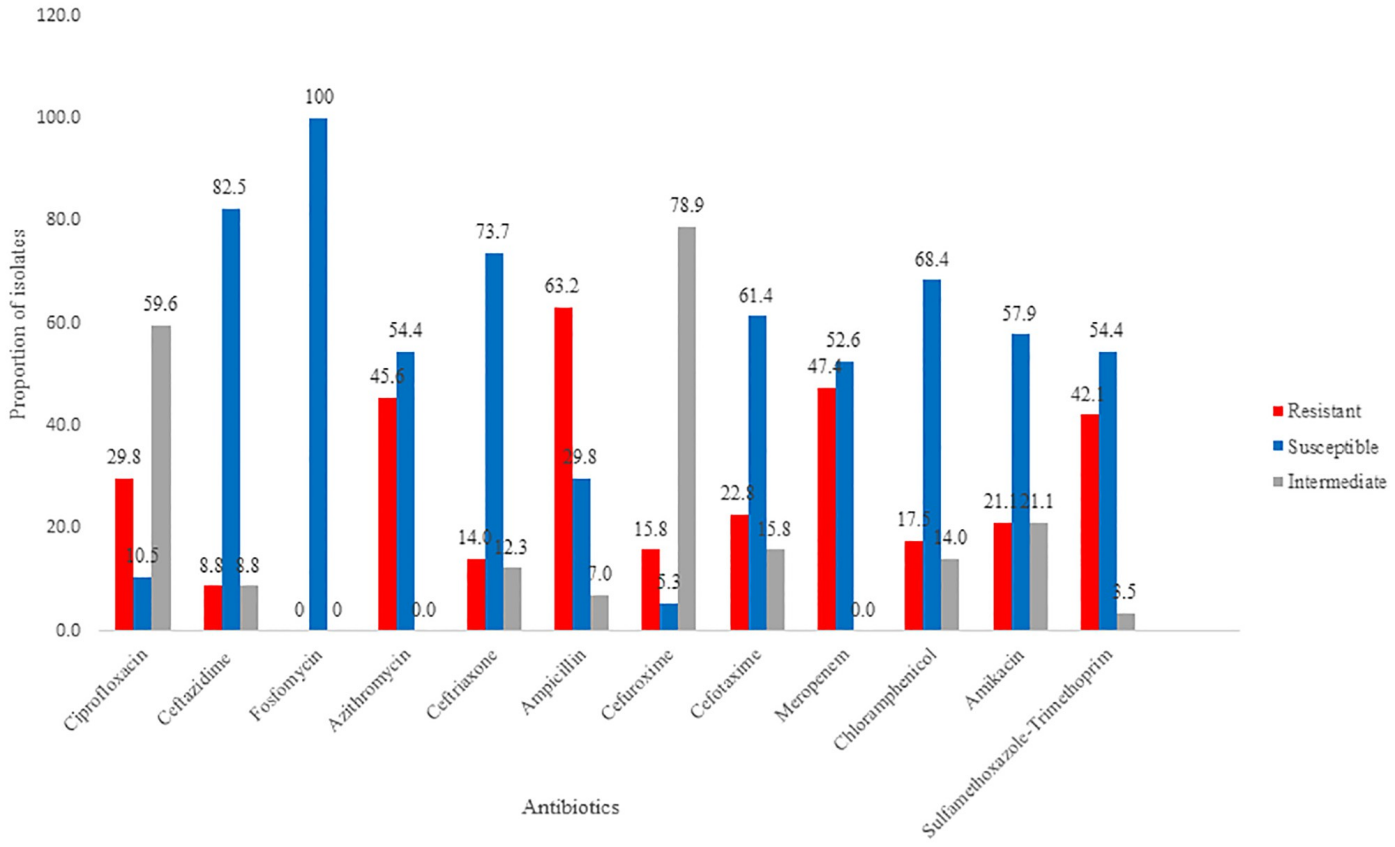
Antibiotic resistance amongst the *Escherichia coli* isolates. Percentages of resistant, susceptible and intermediate isolates are shown in red, blue, and grey color respectively.

In general, *E*. *coli* isolates demonstrated varying levels of resistance to the antibiotics against which they were tested. Of the 57 *E*. *coli* isolates tested, 75.4% (43/57) showed resistance to at least one of the 12 antibiotics they were tested against. A total of 19%, 12%, 9%, 8% and 7% of the isolates observed to be MDRs were resistant to three, five, nine, nine and six antibiotics, respectively (S1 Fig). Three isolates from OD and one from hospital effluent (HE) were observed to be resistant to nine antibiotics. One isolate each from HE and OD, and two from KL were observed to be resistant to eight antibiotics (S2 Fig). Generally, resistance ranged from 8.8% (CAZ) to 63.2% (AMP). Most isolates (over 40%) were resistant to AMP 63.2%(36/57), MEM 47.4% (27/57), AZM 45.6%(26/57), and SXT 42.1%(24/57). Some of the isolates (below 40%) showed resistance to CIP 29.8%(17/57), CAZ 8.8%(5/57), CRO 14%(8/57), C 17.5%(10/57), CTX 22.8%(14/57), AK 21.1%(12/57), and CXM 15.8%(10/57). All isolates were susceptible to fosfomycin (100%) (S3 Table; Fig 2). Isolates from OD showed high resistance rates ranging from 9.3% to 41.9%, followed by isolates from HE, with rates ranging from 2.3% to 16.3%; and from 2.3% to 9.3% for KL and WWTP samples (S4 Table; S3 Fig). A significant difference in antibiotic resistance of the isolates was observed between the sites (p < 1.72E-4; 95% CI).

The resistance profiles AMP-SXT, AMP-MEM, AMP-SXT-CIP, AZM-MEM, and AZM-MEM-AMP-C-SXT were observed in both seasons (Table 1). Some isolates were resistant to a maximum of nine and eight antibiotics with the following observed resistance patterns: CIP-CAZ-AZM-AMP-CRO-CTX-MEM-AK-SXT, CIP-AZM-CRO-AMP-CTX-MEM-C-AK-SXT, CIP-AZM-CXM-CRO-AMP-MEM-C-AK, and CIP-AZM-CRO-AMP-CXM-CTX-MEM-C (S4 Fig). Of the 43 *E*. *coli* isolates (43/57), 36(84%) were resistant to beta-lactam antibiotics, whereas 14 (33%) were ESBL producers. The resistant isolates were mostly found in water sources 49%(28/57) versus sediment sources 26%(15/57). Table 1 shows the season, source, resistance profiles, and ESBL-producing status of the *E*. *coli* isolates.

**Table 1:** Season, source, antibiograms, and extended-spectrum beta-lactamase (ESBL)-producing status of the *Escherichia coli* isolates.

No	Season	Site	Site code	Source	Isolate	Antibiogram	ESBL
1	Dry season	Odaw downstream	ODDS1	water	D3-1	AMP+SXT+CIP	-
2		Odaw upstream	ODUS1	water	D9-1	AMP+C+SXT+AZM	+
3		Korle sewage	KLSW1	water	D2-1	AMP+CXM+CTX+SXT+CIP+ AZM+CRO	+
4		Odaw downstream	ODDS2	water	D4	AMP+SXT	-
5		Odaw upstream	ODUS2	water	D8	AMP+SXT	-
6		Hospital effluent	LH	water	E2-1	AMP+CXM+CTX+C+SXT+ AZM+CRO	+
7		Korle Lagoon MS	KLMS2	water	C11	AZM	-
8		Hospital effluent	37 MH	water	E12	AMP+CXM+SXT+AZM	+
9		Hospital effluent	KBTH	water	E11	AMP+SXT	-
10		Korle sewage	KLSW2	water	F8	AZM+MEM+AMP+C+SXT	+
11		Korle sewage	KLSW2	water	F7	AZM+MEM+AMP+SXT	+
12		Odaw midstream	ODMS3	water	B7	CIP+AZM+AMP+MEM	-
13		Hospital effluent	37 MH (1.2)	sediment	D5	CIP+AZM+AMP+MEM+C+SXT	+
14		Hospital effluent	37 MH (1.2)	sediment	D3	CIP+ AZM+ CRO+ AMP+ CTX+ MEM+ C+ AK+ SXT	+
15		Hospital effluent	37 MH (W)	water	F4	CIP+ AZM+ CAZ+ AMP+ CTX+ MEM+ AK+ SXT	+
16		Korle Lagoon DS	KLDS1	water	F1	CIP+ CAZ+ AZM+ AMP+ CXM+ CTX+ MEM+ AK	-
17		Odaw midstream	ODMS3	water	F9	CIP+ CAZ+ AZM+ CRO+ AMP+ CXM+ CTX+ AK+ SXT	+
18		Odaw midstream	ODMS3	sediment	C7	CIP+ CAZ+ AZM+ CRO+ AMP+ CXM+ CTX+ MEM+ AK	+
19		Odaw downstream	ODDS1	water	C6	AMP+ MEM	-
20		Odaw midstream	ODMS3.2	water	D8	AZM+ AMP+ CXM+ CTX+ MEM	+
21		Hospital effluent	37 MH (1.2)	sediment	D12	CIP+ AZM+ AMP+ MEM+ AK+ SXT	+
22		Odaw downstream	ODDS1	water	C5	AZM+ CTX+ MEM	-
No	Season	Site	Site code	Source	Isolate	Antibiogram	ESBL
23		Korle Lagoon DS	KLDS1	water	F3	CIP+ AZM+ AMP+ CXM+ CTX+ MEM+ C+ AK	-
24		Korle sewage	KLSW2	water	F6	AMP+ MEM	-
25		Odaw midstream	ODMS3.2	water	D10	AMP+ MEM	-
26		Odaw midstream	ODMS3.2	water	D7	CIP+ AMP+ MEM+ C+ SXT	-
27		Korle Lagoon DS	KLDS2	water	F5	AZM+ AMP+ C+ SXT	+
28		Odaw midstream	ODMS3	water	D9	AMP+ SXT	-
29		Korle Lagoon DS	KLDS1	water	F2	AMP+ SXT	-
30	Wet season	Korle sewage	KLSWIW	water	E4	SXT+AZM	-
31		Korle Lagoon DS	KLDS1W	water	E6	AMP+SXT+CIP	-
32		Odaw upstream	ODUSIW	water	C3	AMP+SXT	-
33		Odaw upstream	ODUS1W	sediment	B9	AZM+MEM+AMP+C+SXT	-
34		Odaw upstream	ODUS1W	sediment	B8	AMP+MEM	-
35		Odaw upstream	ODUS1W	sediment	B10	AZM+MEM	-
36		Odaw upstream	ODUS1W	sediment	B11	CIP+ CAZ+ AZM+ AMP+ CRO+ CTX+ MEM+ AK+ SXT	+
37		Odaw upstream	ODGUS2W*	sediment	C3	AK+ MEM	-
38		Odaw upstream	ODUS2W	sediment	D1	CIP+ AZM+ AMP+ CTX+ MEM+ AK	-
39		Odaw upstream	ODGUS2W*	sediment	B12	CIP+ AZM+ CXM+ CRO+ AMP+ MEM+ C+ AK	-
40		Odaw upstream	ODGUS2W*	sediment	C2	AZM+ MEM	-
41		Odaw upstream	ODGUS2W*	sediment	C1	AZM+ CRO+ AMP+ CTX+ MEM	-
42		Odaw upstream	ODGUS1W	sediment	C11	MEM	-
43		Odaw upstream	ODUS2W	sediment	D2	MEM	-

AMP—Ampicillin, CXM—Cefuroxime, CTX—Cefotaxime, CAZ—Ceftazidime, CRO—Ceftriaxone, CIP—Ciprofloxacin, AZM—Azithromycin, AK—Amikacin, MEM—Meropenem, C—Chloramphenicol, AK—Amikacin, SXT-Sulfamethoxazole-Trimethoprim ESBL—Extended spectrum beta-lactamase, ‘-’ means negative, ‘+’ means positive.

### Determination of the multiple antibiotic resistance index

The Multiple Antibiotic Resistance (MAR) index is a means of source tracking antibiotic-resistant isolates. The MAR index was determined by dividing the number of antibiotics to which an isolate is resistant to (a) by the total number of the antibiotics used in the study (b): a/b (S4 Table). The MAR indexes of the 43 *E*. *coli* isolates ranged from 0.08 to 0.8 with the predominant MAR index being 0.2 in 14 (33%) isolates. Out of the total isolates, 40 (93%) had an MAR index ≥0.2.

### Genome characteristics of sequenced *Escherichia coli* isolates

A total of 25 *E*. *coli* isolates (25 of 43) showing resistance to three or more antibiotics [24], representing 44% (25/57) of isolates collected from all five sampling sites, were sequenced. The genome characteristics of the sequenced *E*. *coli* isolates, including the total assembled genome, the GC content, N50, and total number of contigs, are also shown in S5 Table.

### Sequence types and phylogroups

The 25 isolates were classified into twenty sequence types (STs; Table 2, S5 Fig). All sample sources exhibited a diversity of STs: ST1662 (n = 2, 8%), ST21 (n = 2, 8%), ST167 (n = 2, 8%), and ST866 (n = 2, 8%). Five isolates were assigned to novel STs: ST14531 (n = 2) belonging to the ST446 clonal complex (CC), ST14536, ST14537, and ST14538 (ST226 clonal complex). Fifteen sequence types were singletons (Table 2).

**Table 2:** Sequence types (STs), phylogroups, resistance genes, virulence genes, and plasmids found in the *Escherichia coli* isolates.

Site	Isolate	ST(ESBL)	Phylogroup	Fum	Fim	Virulence genes	Acquired Resistance Genes
Hospital effluent	E2_S39_L001	700 (+)	B2	fumC108	none	afaA, afaB, afaC, afaD, afaE, chuA, fyuA, gad, hra, iha, irp2, iss, iucC, iutA, kpsE, ompT, sitA, tcpC, terC, traT, usp, vat, yfcV	aac(3)-IId, aadA2, aph(6)-Id, mph(A), sul2, sul1, sitABCD, tet(B), aadA1, blaCTX-M-15, blaTEM-1B, catA1, qacE, dfrA12, aph(3’’)-Ib, dfrA1
Hospital effluent	E12_S41	14538 (+)	A	fumC27	fimH41	yehD, yehC, yehB, yehA, csgA, terC, nlpI, fimH, terC, clpK1, hlyE, AslA	sul2, tet(A), aph(3’’)-Ib, dfrA14, blaTEM-1B
Hospital effluent	D5_S152	866 (+)	B1	fumC6	none	lpfA, fdeC, yehA, yehD, yehB, yehC, hha, terC, capU, traT, anr, hlyE, gad, nlpI, csgA	aadA1, aph(6)-Id, qnrS1, sul2, tet(B), blaOXA-181, blaOXA-1, blaTEM-35, catA1, dfrA1
Hospital effluent	D3_S43	218 (+)	A	fumC29	none	yehC, yehD, yehA, yehB, fdeC, hlyE, csgA, AslA, nlpI, terC, fimH	blaTEM-1B, qnrS1, tet(A), mph(A), dfrA14, sul2
Hospital effluent	F4_S157	202 (+)	A	fumC11	none	yehA, gad, nlpI, terC, hlyE, yehB, yehC, yehD, csgA	sul2, aph(6)-Id, aph(3’’)-Ib, qnrS1, blaTEM-1B, dfrA14
Hospital effluent	D12_S164	342 (+)	A	fumC11	fimH41	gad, yehB, yehC, yehA, yehD, AslA, hlyE, csgA, terC, nlpI, fdeC, fimH	mph(A), dfrA14, sul2, blaTEM-1B
Korle Lagoon DS	F1_S159	196	B1	fumC19	fimH31	fimH, yehA, yehC, yehB, yehD, shiB, gad, csgA, hlyE, hha, ompT, lpfA, nlpI, fdeC, terC	tet(A), fosA7
Korle Lagoon DS	F3_S150	37	A	fumC11	fimH23	yehD, yehB, yehA, yehC, clpK1, fdeC, terC, hlyE, fimH, gad, iss, csgA, AslA	-
Korle Lagoon DS	F5_S151	492 (+)	A	fumC7	fimH25	fdeC, gad, yehC, yehD, yehB, yehA, terC, anr, nlpI, hlyE, csgA, fimH	aph(6)-Id, qnrS1, sul2, blaTEM-1C
Korle Lagoon DS	E6_S42	446	A	fumC11	fimH23	gad, hlyE, terC	aph(6)-Id, sul2, tet(A)
Korle sewage	D2_S40_L001	167 (+)	A	fumC11	none	fyuA, gad, hlyE, irp2, terC, traT	tet(B), erm(B), mph(A), blaCTX-M-15, catA1, qacE, sul1, dfrA12, aadA2
Korle sewage	F8_S165	14536 (+)	B1	fumC4	fimH32	terC, gad, yehA, yehC, yehD, yehB, hlyE, fimH, fdeC, lpfA, nlpI, csgA	sul2, tet(B), blaTEM-1B, catA1
Site	Isolate	ST (ESBL)	Phylogroup	Fum	Fim	Virulence genes	Acquired Resistance Genes
							qnrS1, aph(6)-Id, aph(3’’)-Ib, sul2, blaTEM-1B, aadA1, qacE, sul1, tet(B), dfrA14
Korle sewage	F7_S155	390 (+)	A	fumC11	fimH54	fimH, AslA, csgA, hlyE, hha, terC mrkA:ABW83989, yehC, yehA, yehB, gad, astA, irp2, fyuA	qnrS1, aph(6)-Id, aph(3’’)-Ib, sul2, blaTEM-1B, aadA1, qacE, sul1, tet(B), dfrA14
Odaw downstream	C5_S153	1972	A	fumC196	fimH305	csgA, gad, fimH, clpK1, nlpI, terC, yehC, yehD, hlyE, etsC	-
Odaw downstream	B7_S168	21	B1	fumC4	fimH121	Iss, gad, terC, hlyE, csgA, lpfA, yehD, yehA, yehC, yehB, nlpI, fdeC, fimH	aph(6)-Id, aph(3’’)-Ib qnrB19, tet(B)
Odaw midstream	F9_S161	167 (+)	A	fumC11	none	fyuA, irp2, yehA, yehB, yehD, yehC, hha, csgA, terC, gad, hlyE, nlpI, AslA	tet(B), catA1, mph(A), qepA4, qacE, sul1, dfrA12, aadA2, blaCTX-M-15
Odaw midstream	C7_S158	1662	B1	fumC23	fimH38	yehB, yehA, yehC, yehD, gad, lpfA, hlyE, csgA, fdeC, terC, nlpI, fimH	-
Odaw midstream	D8_S149	14537 (+)	A	fumC56	fimH54	yehA, yehB, fimH, yehD, yehC, terC, colE7, hha, hlyE, gad, nlpI, csgA, clpK1	qnrB4, blaTEM-1B sul2, tet(A), aph(6)-Id aph(3’’)-Ib, qacE sul1, blaDHA-1
Odaw midstream	D7_S154	19	B1	fumC29	fimH38	etsC, gad, hlyE, iss, IpfA, terC, mrkA:ABW83989, fdeC, yehB, yehC, yehA, yehD, nlpI, clpK1, fimH, csgA, anr	sul2, tet(B), catA1, aph(6)-Id
Odaw midstream	D9_S44	866 (+)	B1	fumC6	none	capU, fyuA, gad, hlyE, irp2, IpfA, terC	tet(B), blaTEM-1B sul2, aph(6)-Id aph(3’’)-Ib, mph(A) catA1, blaOXA-1, aadA1, dfrA1
Odaw upstream	B9_S166	845 (+)	A*	fumC4	fimH60	yehB, yehC, yehA, yehD, terC, nlpI, hlyE, csgA, gad, fimH, lpfA, fdeC	-
Odaw upstream	B11_S156	21	B1	fumC4	fimH121	terC, hlyE, csgA, fdeC, yehA, yehD, yehC, yehB, hha, gad, fimH, lpfA, nlpI	tet(A), dfrA14, blaTEM-1B
Odaw upstream	D1_S162	1662	B1	fumC23	fimH38	gad, hlyE, IpfA, terC, fimH, fdeC, csgA, nlpI, yehD, yehB, yehC, yehA	-
Odaw upstream	B12_S163	14531	B1	fumC19	fimH31	gad, hlyE, iss, IpfA, sitA, terC, csgA, nlpI, yehD, yehB, yehC, yehA, fimH, fdeC	tet(A), sitABCD
Odaw upstream	C1_S167	14531	B1	fumC19	fimH31	csgA, iss, sitA, hlyE, fimH, yehD, yehA, yehC, yehB, terC, nlpI, gad, fdeC, lpfA	tet(A), sitABCD,

The most common phylogroup identified among the collection was A (13; 52%), followed by B1 (11; 44%), and B2 (1; 4%) (S6 Fig). Isolates from OD belonged to phylogroups A (4; 16%) and B1 (8; 32%). Meanwhile, phylogroup A was the most common isolate from KL (3; 12%), WWTP (4; 16%), and HE (2; 8%) (S7 Fig). Interestingly, only one isolate from HE belonged to phylogroup B2, which is usually associated with pathogenicity (Table 2). Of the 14 EBSL isolates observed, eight (57%) belonged to phylogroup A, five (36%) belonged to B1, and one (7%) belonged to B2, and were mostly MDR.

### Antibiotic-resistant genes of *Escherichia coli* isolates

Thirty-seven different types of ARGs, totaling 128 ARGs in all, were found among the *E*. *coli* isolates (21/25) observed across all studied sources (Table 2; S8 Fig). The dominant ARGs identified comprised the sulfonamide resistance gene *sul2* (12/128; 9.4%), aminoglycoside resistance gene *aph(6)-Id* and tetracycline resistance gene *tetB* (9/128; 7% each), chloramphenicol resistance gene *catA1* (8/128; 6.3%), sulfonamide resistance gene *sul1* and folate pathway antagonist *dfrA14* (7/128; 5.4% each), quinolone resistance gene *qnrS1*, and macrolide resistance gene *mph(A)* (6/128; 4.7% each). Additional genes included *aac(3)-IId*, *ant(3’’)-Ia*, *aph(3’)-Ia*, *aadA1*, *aadA2* (aminoglycosides), *qepA4*, *qnrB1*, *qnrS1*, *qnrB19* (quinolones), *dfrA7*, dfrA12 (folate pathway antagonist), *floR* (amphenicols), *qacE* (biocide resistance), *sitABCD* (hydrogen peroxide), and *erm(B)* (macrolides, lincosamides, and streptogramin b).

No ARGs included in the ResFinder databases were identified in four isolates (16%), two from OD and two from the KL. The fosA7 gene, which mediates resistance to fosfomycin, was identified in one *E*. *coli* isolate, but all *E*. *coli* isolates were susceptible to fosfomycin. Further genomic investigations are required to understand the resistance mechanisms in these environmental isolates.

The β-lactamase genes identified comprised the class A (Ambler classification) EBSL genes, *blaTEM-1B* 7.8% (10/128), *blaCTX-M-15* 2.3% (3/128), *blaTEM-1C* 1.7% (2/128), and *blaTEM-35* genes. Additionally, singletons such as the class D oxacillinase genes *blaOXA-1*, *-181* and the class B ampC β-lactamase gene: blaDHA-1 were identified. One isolate (C7) phenotypically showed ESBL activity but, not harbour ESBL genes. Further genomic analysis is needed to understand the resistance mechanism employed by this strain. ESBL-producing activity was observed among 12 STs (48%; 12/25), which demonstrated a wide range of resistance to a β-lactam antibiotic, cephalosporins, and other antibiotic classes such as quinolones, and carbapenems (Table 2). The dominant β-lactamase genes were observed in isolates belonging to STs ST866 21%(4/19), ST700 11% (2/19), and ST 167 11%(2/19).

Mutations in the quinolone resistance-determining regions (QRDRs) in chromosomal DNA gyrase (gyrA and gyrB) and DNA topoisomerase IV (parC and parE) genes were investigated for all isolates. The gyrA gene (S83L, D87N), parC gene (S80I), and parE gene (S458A) all exhibited mutations in isolate D2_S40_L001, conferring chromosomal mutations associated withfluoroquinolones resistance. The isolate (E2_S39_L001) had a mutation in the gyrA gene (S83L). Isolate D7 had three mutations in pmrA (E147K), folP (V263L), and parC (E62K) genes; however, the mutations are not known. Isolate E12 had a mutation in parC (E62K).

### Genetic environment of ARGs of beta-lactamase-producing isolates

The majority of β-lactamase-producing strains were observed to possess ARGs associated with MGEs, including class 1 integrons, plasmids, insertion sequences, and transposons. These results highlight the genomic adaptability of the isolated strains (Table 3). The *blaTEM* gene exhibited a high frequency of association with a protein belonging to the recombinase family. The *Tn3* family transposon was identified as the most prevalent transposon and was primarily linked to quinolone, sulfonamide, tetracycline, and trimethoprim. Furthermore, the presence of the *IS91* insertion sequence was commonly detected and found to be correlated with resistance to sulfonamide, trimethoprim, and quinolone antibiotics. The presence of the *qacEΔ1* gene, which confers resistance to disinfectants, was linked with the sulfonamide resistance gene *sul1* and the aminoglycoside resistance genes *aadA2* (specifically for streptomycin and spectinomycin) in four isolates (F7, E2, F9, and D2) and only the *sul1* gene in one isolate (F8) from HE, WWTP, and OD. Additionally, *qacEΔ1* gene was associated with a PMQR gene (*qepA4*) encoding a 14-transmembrane-segment efflux pump belonging to the major facilitator superfamily (MFS), associated with resistance to ciprofloxacin. The ARGs and MGEs found in the *E*. *coli* isolates exhibited a high degree of similarity (ranging from 98% to 100%) with the target sequences stored in the GenBank database. Most hits corresponded to various *E*. *coli* plasmids, along with plasmids linked to other members of the Enterobacteriaceae family. One isolate (D12) contained the plasmid *pRiA4b ORF-3* family protein, which exhibited similarity to the protein encoded by *ORF-3* (Q44206) present on the plasmid *pRiA4* in the bacterium *Agrobacterium rhizogenes*. This plasmid has been reported to play a significant role in the development of tumours at wound sites in plants infected by the bacterium [37].

**Table 3:** Antibiotic resistance genes and associated genetic elements in β-lactamase-producing environmental *E*. *coli* isolates.

Strain (MLST)	Contig	Synteny of resistance genes and MGEs	Plasmid/chromosomal sequence with closest nucleotide homology (accession number
ECD3010	75	transposase zinc-binding domain-containing protein:blaTEM-1: recombinase family protein	*Escherichia coli* EC261 plasmid pEC261_3 (AP027420.1)
	61	recombinase family protein::IS91-like ISVsa3::IS110-like IS5075:Arm:Sul2	*Escherichia coli* plasmid p33 (MT077884.1)
	60	IntI1:dfrA14: MobC::: IS6-like IS6100: mphR(A): mrx(A): Mph(A) family macrolide 2’-phosphotransferase	*Escherichia coli* DETEC-P169 plasmid pDETEC1 (CP116167.1)
	41	Tn3-like TnAs1::EamA:tet(A): TetR(A):: plasmid pRiA4b ORF-3 family protein:: ISKra4-like ISKpn19:: QnrS1	*Escherichia coli* EC20-4B-2 plasmid pEC20-4B-2-1 (AP026938.1)
ECE12018	50	IS5-like IS903B:Tn3-like Tn3::blaTEM-1: TetR(A): tet(A):::: Tn3-like TnAs1:Sul2: dfrA14: APH(3’)::: IS91:::::: IS1-like transposase	*Escherichia coli* 15OD0495 plasmid p15ODMR (MG904997.1)
ECF8024	87	Sul2: APH(3’’)-Ib: APH(6)-Id: IS91:blaTEM-1: recombinase: Tn3-like Tn3: IS3: QnrS1	*Shigella flexneri Y* RC960 plasmid pRC960-1 (KY848295.1)
	132	catA1	*Escherichia coli* AHM21C2872I chromosome (CP104629.1)
	117	Sul1:QacE delta 1: ANT(3’’)-Ia	*Escherichia coli O157*:*H7* strain K1516 chromosome (CP049612.1)
	119	Transposase: tet(B): tetR(B)	*Escherichia coli strain RHB20-E3-C03 plasmid (CP099204*.*1)*
	100	IS1::: IS6-like IS6100::MobC: dfrA14	*Klebsiella pneumoniae strain 24169 plasmid p24169-KPC (MN891676*.*1)*
	3	MFS::EmrR: EmrA: EmrB	*Escherichia coli strain OSUCMP42NDM chromosome (CP087578*.*1)*
ECF9025	83	IS91 family transposase::fluoroquinolone efflux MFS transporter QepA4	*Escherichia coli strain KFS-D29 plasmid pKFS-D29_1 (CP125007*.*1)*
	71	sul1:: QacE delta 1:AadA::dfrA12	*Escherichia coli plasmid p65 (MT077888*.*1)*
	24	blaCTX-M-15:: Tn3-like Tn3 family transposase	*Escherichia coli strain MRSN346355 (CP018121*.*1)*
	86	erm(B)	*Escherichia coli strain 5M plasmid pISV_IncFII_NDM-5 (MN218686*.*1)*
ECD5011	74	sul2: aph(3’’)-Ib: APH(6)-Id: IS91 family transposase:::blaTEM-190: recombinase family protein	*Escherichia coli strain 1EC213 plasmid pEC213_1-OXA-181 (CP061102*.*1)*
	79	tetC: tet(B): TetR(B)	*Escherichia coli strain G12 plasmid pG12a (CP129273*.*1)*
	70	Transposase: QnrS1: recombinase family protein:: plasmid pRiA4b ORF-3 family protein: recombinase family protein: ISKra4-like ISKpn19 family transposase:: EreA family erythromycin esterase: blaOXA-181:: Tn3 family transposase	*Escherichia coli strain 142 plasmid p142_A-OXA181 (CP048338*.*1)*
	71	catA1:: Tn3-like TnAs3 family transposase: recombinase family protein:: intI1:blaOXA-1: aadA1	*Escherichia coli strain 1EC213 chromosome (CP061101*.*1)*
	5	TnsD family Tn7-like transposition protein: Tn7-like element transposition protein TnsE: IS256 family transposase::::dfrA1: intI2	*Escherichia coli strain 61 chromosome (CP048326*.*1)*
ECE2016	59	tetR(B): tet(B): tetracycline efflux MFS transporter Tet(B):: IS4-like ISVsa5 family transposase	*Shigella flexneri strain FDAARGOS_691 chromosome (CP054913*.*1)*
	61	catA1:: Tn3-like TnAs3 family transposase	*Shigella flexneri strain FDAARGOS_691 chromosome (CP054913*.*1)*
	54	aac(3)-IId::::: IS1182-like ISCfr1 family transposase	*Escherichia coli strain EcPF7 chromosome (CP054232*.*1)*
	49	intI1: dfrA12: aadA2: QacE delta 1: Sul1::::: IS6-like IS6100 family transposase: mphR(A): mrx(A):Mph(A)	*Escherichia coli TUM18530 DNA (AP023190*.*1)*
	60	sul2: aph(3’’)-Ib: APH(6)-Id	*Escherichia coli O2*:*K1*:*H4 strain APEC E19019 chromosome (CP126937*.*1)*
	25	intI2: dfrA1:sat2: aadA1::: IS256 family transposase: Tn7-like transposition protein TnsE: TnsD family Tn7-like transposition protein:: DDE-type integrase/transposase/recombinase	*Escherichia coli strain FDAARGOS_536 chromosome (CP033762*.*1)*
	87	blaTEM-1	*Enterobacter roggenkampii strain 2017-45-51-04-01 plasmid p2017-45-51-04-01_1 (CP109730*.*1)*
	50	blaCTX-M-15: IS1380-like ISEcp1 family transposase: transposase domain-containing protein: IS110-like ISEc45 family transposase: transposase	*Shigella sonnei strain S17BD05200 chromosome (CP110404*.*1)*
ECF7023	66	QnrS1: IS3 family transposase: Tn3-like Tn3 family transposase: recombinase family protein:blaTEM-1: IS91 family transposase: APH(6)-Id: aph(3’’)-Ib: sul2:: IS110-like IS5075 family transposase: Tn3 family transposase	*Shigella flexneri Y strain RC960 plasmid pRC960-1 (KY848295*.*1)*
	99	Integrase: aadA1: QacE delta 1: sul1	*Escherichia coli O157*:*H7 strain K1516 chromosome (CP049612*.*1)*
	76	IS4-like ISVsa5 family transposase:::::tetR(B):tet(B):transposase	*Salmonella enterica subsp*. *enterica serovar Typhimurium strain ST45 chromosome (CP050753*.*1)*
	85	IS1 family transposase:::: IS6-like IS6100 family transposase: mobC: dfrA14: intI1:: recombinase family protein	*Klebsiella pneumoniae subsp*. *pneumoniae strain ST2017*:*950142398 plasmid p18-43_01 (CP023554*.*1)*
B11_S156	41	recombinase family protein: ISKra4-like ISKpn19 family transposase: recombinase family protein: QnrS1: IS3 family transposase: Tn3-like Tn3 family transposase: recombinase family protein:blaTEM-1: recombinase family protein: APH(6)-Id: APH(3’’)-Ib: APH(3’’)-Ib: IS110-like IS5075 family transposase	*Shigella flexneri Y strain RC960 plasmid pRC960-1 (KY848295*.*1)*
	43	IS1 family transposase: tyrosine-type recombinase/integrase:: Tn3-like TnAs1 family transposase::: tet(A): tetR(A):: Tn3-like TnAs1 family transposase	*Escherichia coli strain CFSAN061766 plasmid pCFSAN061766 (CP042872*.*1)*
	51	recombinase family protein: class 1 integron integrase IntI1: dfrA14	*Escherichia coli strain CFSAN061766 plasmid pCFSAN061766 (CP042872*.*1)*
ECD8013	143	QnrB4	*Klebsiella pneumoniae strain AHM8C161BI plasmid pHNAH8161B-2 (CP104605*.*1)*
	58	sul2: Tn3-like TnAs1 family transposase::: tet(A): tetR(A):blaTEM-1: recombinase family protein: Tn3 family transposase	*Enterobacter hormaechei strain Eho-2 plasmid pEcl2-1 (CP047758*.*1)*
	135	aph(3’’)-Ib: APH(6)-Id	*Escherichia coli strain AH25 plasmid pAH25-2 (CP055258*.*1)*
	88	blaDHA:::: sul1	*Klebsiella quasipneumoniae strain EF262 plasmid p4 (CP092516*.*1)*
ECD12015	70	recombinase family protein:blaTEM-1	*Escherichia coli strain TUM13773 plasmid pMTY13773_IncF (CP128913*.*1)*
	56	recombinase family protein:: IS91-like ISVsa3 family transposase:: sul2:: IS110-like IS5075 family transposase	*Escherichia coli plasmid p33*, *complete sequence (MT077884*.*1)*
	55	Mph(A): mrx(A): mphR(A): IS6-like IS6100 family transposase:: MobC: dfrA14: intI1	*Escherichia coli strain 165 chromosome*, *complete genome (CP020509*.*1)*
	37	Transposase: QnrS1: recombinase family protein: ISKra4-like ISKpn19 family transposase: recombinase family protein: plasmid pRiA4b ORF-3 family protein: tetR(A): tet(A)::: Tn3-like TnAs1 family transposase	*Escherichia coli 2017*.*04*.*03CC plasmid p20170403CC-1 DNA (AP025206*.*2)*
ECF4021	56	Tn3 family transposase: IS110-like IS5075 family transposase:: sul2: aph(3’’)-Ib: APH(6)-Id: IS91 family transposase:blaTEM-1: recombinase family protein: Tn3-like Tn3 family transposase: IS3 family transposase: QnrS1:ISKra4-like ISKpn19 family transposase: recombinase family protein: plasmid pRiA4b ORF-3 family protein	*Escherichia coli strain P21_SE1_04*.*20 plasmid pNDM_2_P21_SE1_04*.*20 (CP085076*.*1)*
	90	dfrA14:intI1:recombinase family protein	*Escherichia coli strain J53-p130 plasmid p130-2 (CP111010*.*1)*
ECD2009	83	tetR(B):tet(B):tetC	*Escherichia coli strain G12 plasmid pG12a (CP129273*.*1)*
	97	erm(B)	*Klebsiella pneumoniae strain ARLG-4803 plasmid pARLG-4803_2 (CP128779*.*1)*
	81	Mph(A):mrx(A):mphR(A):IS6-like element IS6100 family transposase	*Escherichia coli YJ3 plasmid pYJ3-a DNA (AP023228*.*1)*
	23	Tn3-like Tn3 family transposase:blaCTX-M-15	*Escherichia coli strain Iso00041 chromosome (CP095155*.*1)*
	64	catA1:Tn3-like TnAs3 family transposase: recombinase family protein:intI1:dfrA12:: aadA2:QacE delta 1:sul1	*Klebsiella pneumoniae strain 39427 plasmid pKPN39427*.*1 (CP054265*.*1)*
ECD9014	85	tetR(B):tet(B):transposase	*Escherichia coli strain 61 chromosome (CP048326*.*1)*
	79	recombinase family protein:blaTEM-1	*Escherichia coli strain FORC_081 plasmid pFORC_081_3 (CP029060*.*1)*
	68	sul2:aph(3’’)-Ib:APH(6)-Id:IS91 family transposase	*Escherichia coli strain RHBSTW-00503 plasmid pRHBSTW-00503_4 (CP056449*.*1)*
	86	Mph(A):mrx(A):MphR(A)	*Providencia huaxiensis strain WCHPr000369 plasmid pIMP4_000369 (CP031121*.*1)*
	63	blaOXA-1:intI1::recombinase family protein: Tn3-like TnAs3 family transposase::catA1	*Salmonella enterica subsp*. *enterica serovar Typhimurium strain ST53 chromosome (CP050745*.*1)*
	125	aadA1	*Escherichia coli strain AH01 chromosome (CP055251*.*1)*
	9	intI2:dfrA1	*Escherichia coli strain Z0117EC0040 chromosome (CP098211*.*1)*
ECF5022	53	blaTEM-1C:recombinase family protein: Tn3-like Tn3 family transposase	*Klebsiella pneumoniae strain GN4542 plasmid pGN4542-1 (CP102693*.*1)*
	72	sul2:aph(3’’)-Ib:aph(3’’)-Ib	*Proteus mirabilis strain MPE5139 chromosome (CP053684*.*1)*
	78	QnrS1:QnrS1	*Escherichia coli strain 1782_2_EMB plasmid p1782_2_EMB (OQ078085*.*1)*

### Virulence-associated genes

Virulome analysis showed a total of 178 VAGs comprising 41 different genes across the 25 *E*. *coli* isolates. All isolates carried between three to 23 VAGs. Seventy (39%) VAGs were observed in phylogroup A, followed by B1 85 (48%) and B2 23 (13%). Among the individual isolates, the isolate in phylogroup B2 exhibited the highest VAGs compared to the individual isolates in phylogroups A and B1. The most dominant VAGs observed were *terC* 14% (25/178), followed by *gad* and *hylE* 13% (24/178 each,) and *IpfA* 7% (12/178) [Table 2; S9 Fig]. Isolates from water recorded the highest number of VAGs 63%(113/178) compared with those from sediment 37%(65/178). In addition, isolates from the OD recorded the highest VAGs 49%(87/178), followed by isolates from hospital effluent 33%(58/178), sewage 10%(18/178), and the KL 8%(15/178). Most VAGs were characteristic of the extraintestinal pathogenic *E*. *coli* (ExPEC) pathotype, including different genes encoding adhesins (*fimH*, *afaA*, *afaB*, *afaC*, *afaD*, *afaE*), iron acquisition systems (*fyuA*, *sitA*, *irp2*), toxins (*hlyE*, *sat*, *usp*, *vat*) and protectins (*traT*, *ompT*, *iss*).

### Mobile genetic elements (plasmids, insertion sequences, and integrons)

A total of 59 plasmid replicons comprising 27 different types were recorded in isolates from all studied sources. The predominant plasmid replicons recorded were *Col440I* (10.2%; 6/59), followed by *IncFIB(AP001918)* and *IncY* (6.8%; 4/59) (Table 4; S10 Fig). Nineteen incompatibility (Inc) plasmid replicons were identified with different frequencies, including *IncFIA*, *IncFIA(HI1)*, *IncFIB(AP001918)*, *IncR*, and *IncY*. The majority of isolates (18/59; 31%) harboured the *IncF* (*FIA*, *FIB*, *FIC* and *FII*), *IncH* (9/59; 15%), *IncI* (5/598.5%) and *IncY* (4/59; 7%). Two isolates harbouring the *IncF* groups also harboured the *Incl* and *IncH* groups. Plasmid replicons (19/59, 32%; 11/59, 19%; 10/59, 17%) comprising different frequencies of the *Inc* (F, H, Y and R), Col and p0111 groups were identified in isolates from the OD, HE, SE, and KL, respectively. However, no plasmids were observed in four isolates; three were from OD and one from KL. The majority of isolates (60%; 15/25) had more than one plasmid replicon. A total of 56 different types of ISs and transposons, totaling 228 in all, were identified in this study. A total of 47 IS families of insertion sequences were identified, followed by the *cn*, *MITEEc1*, and *ICEE* families with three and one, respectively. *MITEEc1* (10.5%), *IS3* (7.5%), *IS609* (5.3%), *ISEc30* (4.8%), *ISEc1* (4.4%), *IS102*, *ISEc38*, *ISKpn8* (3.9%), *IS26*, *IS629* (3.1%), *IS5075*, *IS30*, *Iskpn19*, *Tn2* (2.6%), and *ISSen4* (2.2%) were the most dominant insertion sequences and transposons identified in this study. All isolates harbored more than one mobile genetic element. There was no distinct pattern in terms of the replicon type, ST, or source of isolation.

**Table 4:** Distribution of mobile genetic elements/mobilome (insertion sequences, plasmids and transposons).

Isolate	Transposons	Plasmids	Insertion sequences
E2_S39	Tn7	IncQ1, IncFIA, IncFII(pRSB107), Col(BS512), Col440II, Col156, IncFIB(AP001918)	MITEEc1, ISEc42, IS682, ISEc30, ISSfl10, IS6100, ISEc9, ISEc45, ISCfr1, ISKpn24, IS100
E12_S41	Tn6024	IncR	ICEEcoED1a-1, IS629, IS911, ISEc30, ISEsa1, IS421, ISEc38, ISEc1, MITEEc1, ISKpn8, IS629, IS26
D5_S152	Tn6024	ColKP3, IncFIC(FII), IncHI1B, IncFIB(Mar), Col440I, IncFIB(AP001918)	MITEEc1, ISEc30, ISKox3, IS629, IS30, ISKpn24, ISSfl10, IS911, ISKpn19, IS3
D3_S43		IncHI1B(CIT), p0111	IS102, ISKpn19, ISEc38, ISVsa3, MITEEc1, IS5, IS26
F4_S157	Tn2	Col440I	MITEEc1, ISEc78, ISEc38, IS609, ISEc1, ISKpn19, IS5075, ISEc31, ISEc30, ISKpn8, IS421, IS3, cn_1670_ISEc1
D12_S164		IncHI1B(CIT), p0111	ISVsa3, IS6100, ISKpn19, IS609, IS3, IS102, MITEEc1, ISEc1, ISKpn26, ISKpn8, IS421, ISEhe3
F1_S159		IncY	IS3, IS609, MITEEc1, ISEc1
F3_S150		-	ISEhe3, MITEEc1, ISKpn8, IS100, IS26, IS421, IS629, IS3
F5_S151	Tn2	IncFIB(K), Col440I, IncFIA(HI1), IncN, IncR	MITEEc1, ISKpn8, IS903, ISEc30, cn_1419_IS903, IS5075, IS609, ISKpn19, IS421, IS629, IS3
E6_S42	Tn6024	IncL/M(pOXA-48), IncY, Col440II, IncR	IS5075, ISKpn19, MITEEc1, ISEam1, ISEc30, IS102, ISEc1, ISEcl1, ISKpn8, IS5075, IS3, IS30
D2_S40		Col(pHAD28), Col156, IncFIA, IncFIB(AP001918)	MITEEc1, ISKpn24, IS30, ISKox3, IS102, ISEc38, ISSen4, IS421, IS3, IS26
F8_S165	Tn2	IncHI1A, IncHI1B(R27), Col440I	IS6100, ISEc33, MITEEc1, IS640, IS30, ISEcl10, ISSso4, ISEc78, ISKpn21, cn_5620_ISKpn21, ISVsa5, ISSen4, IS5075, IS3, ISEc52, ISKpn26
F7_S155	Tn2	IncR, Col(IRGK), IncHI1B(R27), IncHI1A	IS5075, ISVsa5, IS6100, MITEEc1, ISEc38, ISEc30, IS640, ISKpn8, IS903, ISEc33, ISEc1, cn_1670_ISEc1, IS609, IS102, Tn5403, ISEc30, ISKpn21, ISSen4
C5_S153		IncFIA(HI1), IncFIB(K)	MITEEc1, ISKpn8, IS30, ISEc38, ISSen4, ISPpu12, ISEc78, IS102
B7_S168		Col440I	IS609, IS30, ISEc31, IS609, IS26, ISVsa5, IS3
F9_S161		IncFIB(AP001918), IncFIA	ISEc38, MITEEc1, ISKpn24, IS609, ISEc1, IS102, ISSen4, IS421, ISKox3, IS3, IS26
C7_S158		-	IS609, MITEEc1, IS3
D8_S149		Col156, IncFIA(HI1), IncFII(Yp), IncFIB(pB171), Col440I	ISEhe3, MITEEc1, ISEc30, ISKox3, IS102, ISEc1, Tn5403, ISEc53, ISEc36, IS421
D7_S154		IncHI1A, IncHI1B(R27), IncR	Tn2, MITEEc1, IS100, ISEc30, ISEc33, ISEc38, IS640, ISCfr27
D9_S44	Tn6024	Col(BS512), Col(pHAD28)	ICEEcoED1a-1, IS911, MITEEc1, ISEc30, IS629, IS102, IS629, IS3, IS26
B9_S166		-	MITEEc1, ISKpn8, IS3, ISEc52, ISSen1, ISEc1
B11_S156	Tn2	IncI2, IncFIB(K)	ISEc38, ISEc1, MITEEc1, IS609, IS609,
D1_S162		-	IS609, MITEEc1, IS3
B12_S163		IncY	MITEEc1, IS3
C1_S167		IncY	IS3, MITEEc1

### Phylogenetic analysis of *Escherichia coli* isolates

**Fig 3:**
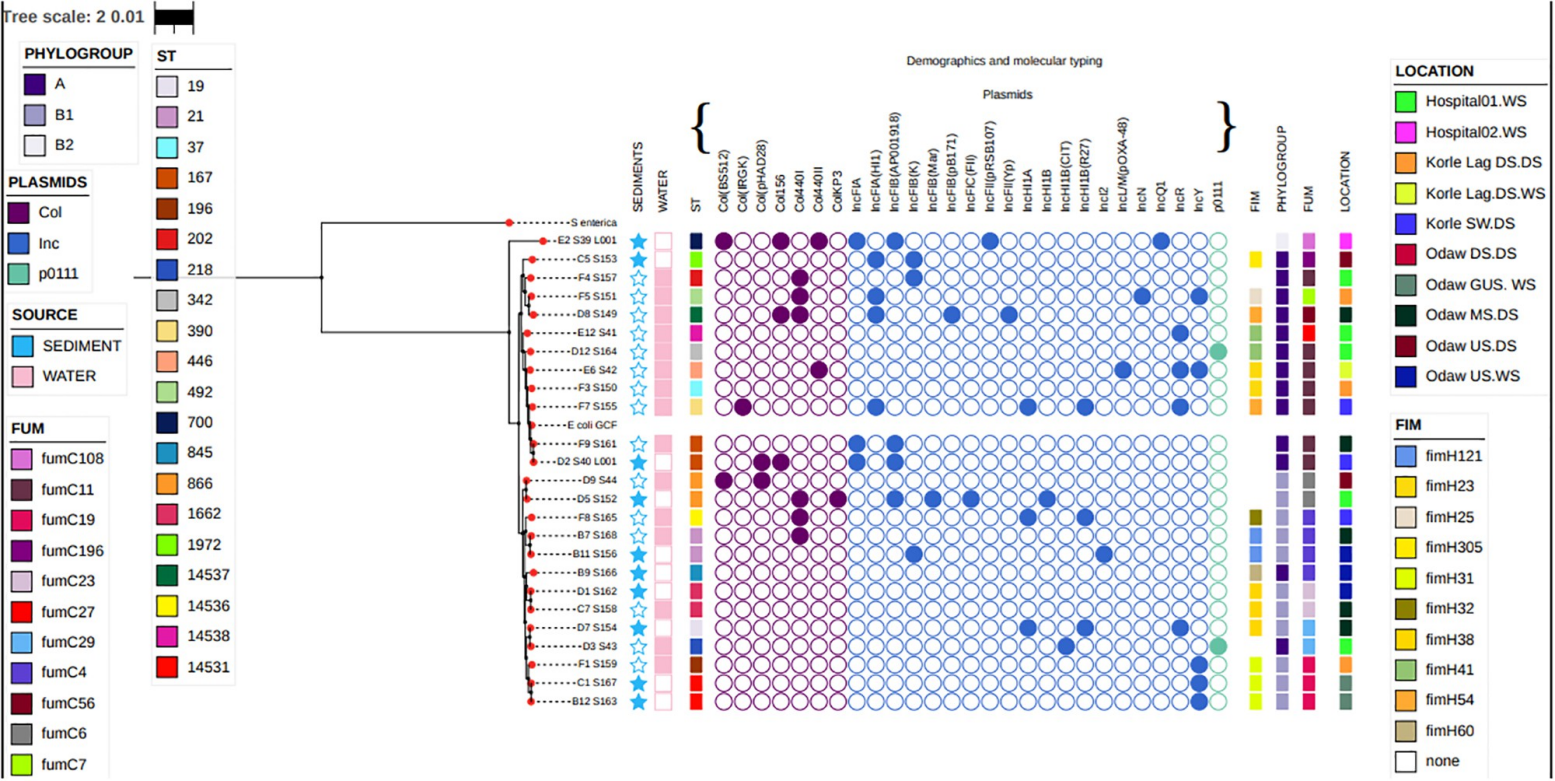
Rooted phylogenetic tree generated on the basis of single-nucleotide polymorphisms (SNPs) of the core genes of 25 *E*. *coli* isolates obtained from environmental sources with reference genome *E*. *coli str*. *K-12 substr*. *MG1655* and *Salmonella enterica subsp*. *enterica serovar Typhimurium str*. *LT2* outgroup. The squares, stars, and circles at the tip the location, sample type, sequence types, fumC and fimH allelesE, and plasmids, respectively.

**Fig 4:**
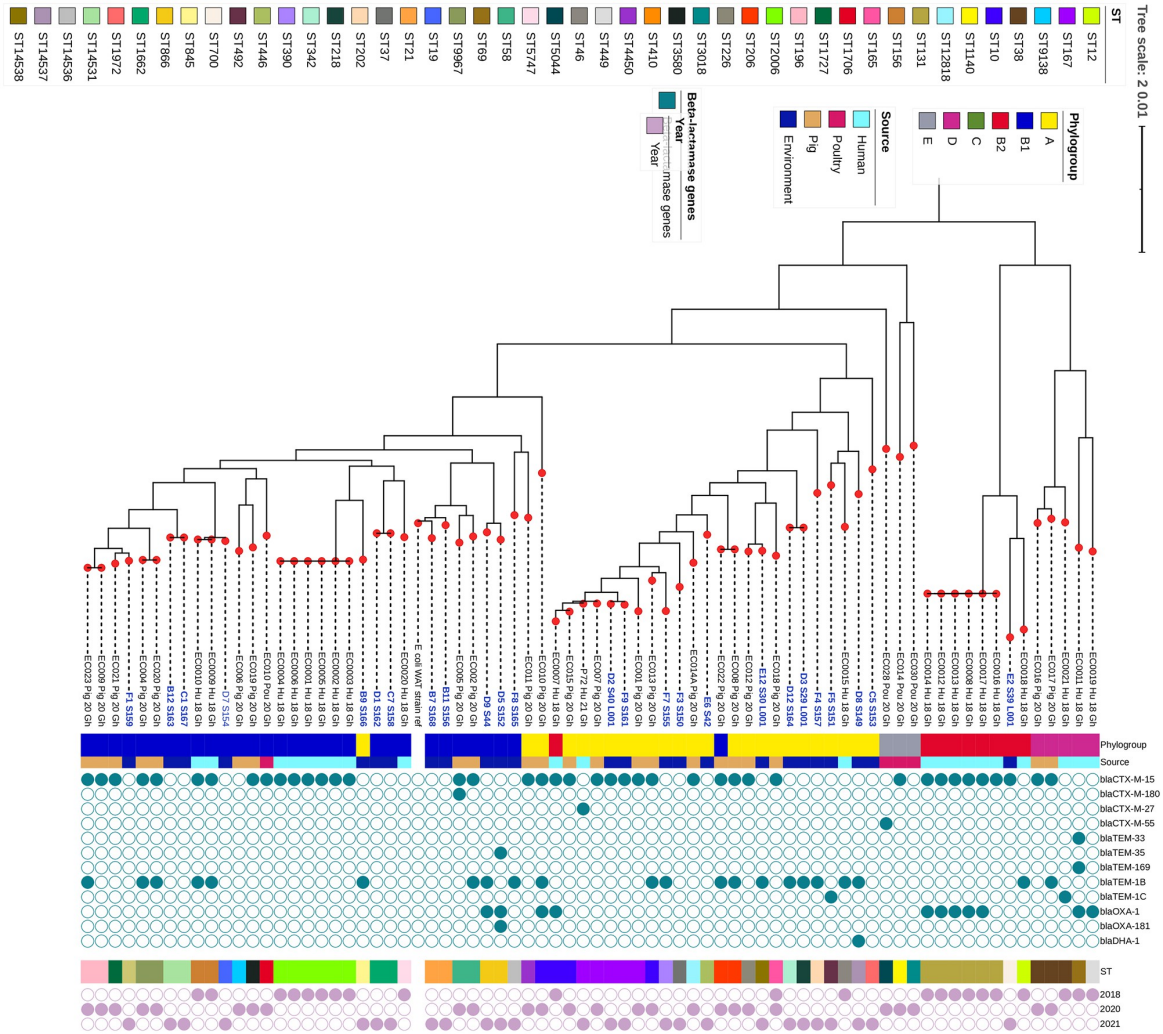
Phylogenetic tree showing the relationship between isolates from this study and Ghanaian isolates from human and animal sources with reference genome *Escherichia coli* str. K-12 substr. MG1655.

Whole genome phylogenetic analysis, in conjunction with the metadata, revealed that isolates clustered into two major clusters, each with subclusters. It was observed that isolates of the same phylogroup clustered together with strains clustering into A, B1, and B2 groups. Clustering was also observed among isolates from the same sample type (water and sediments). Similarly, isolates belonging to the same *Fum* and *Fim* types clustered together (Fig 3). It was observed that the *E*. *coli* strains from this study clustered with *E*. *coli* strains from humans, poultry, and pigs. *Escherichia coli* clustered together based on similarities within the phylogroups. *Escherichia coli* strains from this study also clustered with other non-environmental strains based on their STs, although a great deal of diversity was observed across all the sample sources (Fig 4).

## Discussion

This present study investigated the genomic characteristics of *E*. *coli* isolated from both wastewater and surface water sources in Accra, Ghana. *Escherichia coli* identified in different water sources exhibited diverse genomic characteristics, including ARGs, beta-lactamases, carbapenemases, and MGEs, displaying both diversity and complexity. These genomic features can be horizontally transferred between different bacterial species, thereby facilitating the dissemination and emergence of resistance genes as they converge within the environment. Consequently, the contaminated urban environment serves as an abundant and ever-changing reservoir of ARBs and ARGs. To the best of our knowledge, this study represents one of the most comprehensive descriptive studies conducted in Ghana, in which the genomics of *E*. *coli* originating from these distinct ecological contexts have been extensively examined using WGS.

The study observed the prevalence of resistance to ciprofloxacin, azithromycin, ampicillin, cefuroxime, cefotaxime, meropenem, chloramphenicol, and sulfamethoxazole-trimethoprim in all sources examined. High resistance were particularly noted for ampicillin, meropenem, azithromycin, and sulfamethoxazole-trimethoprim. This finding is consistent with the results of a study conducted in Ghana by Addae-Nuku et al. (2022) [15], which observed a high prevalence of resistance among isolates from hospital wastewater to sulfamethoxazole-trimethoprim, ampicillin, ceftriaxone, and ceftazidime. The results of this study also align with previous research in South Africa [14], documenting predominant resistance to ampicillin (83.3%) and sulfamethoxazole-trimethoprim (75%). Gaşpar *et al*. (2021) [38] reported relatively elevated resistance rates in Romania, specifically to fosfomycin (63%), ampicillin (72%), sulfamethoxazole-trimethoprim (54%), ceftazidime (58%), cefotaxime (28%), ciprofloxacin (36%), and meropenem (4%). An interesting observation in their study is the varying resistance levels to fosfomycin and meropenem compared with 100% susceptibility to fosfomycin and the high resistance to meropenem observed in this study.

The high prevalence of resistance observed in this study may be attributed to several factors, with the primary one being the inadequacy of waste management infrastructure to treat waste before discharge into various locations such as the OD and the KL [15,39]. This results in the contamination of these sources with ARBs, which may outcompete native bacteria over time and disseminate ARGs to other bacterial species through HGT. Other possible factors contributing to the high prevalence observed could be the intense usage of these antibiotics in healthcare settings and the subsequent development of resistance, as previously reported [40–42]. Furthermore, self-medication practices [43] and use of antibiotics in animal husbandry [44] could contribute to the development and spread of ARB in the community. The presence of these factors, coupled with the current challenge of improper waste handling facing the city of Accra, could result in the discharge of resistant bacteria and antibiotic residues into the environment. Moreover, the documented presence of residues from β-lactams, quinolones, cephalosporins, and sulfonamides in environmental sources in Ghana in previous studies [3,45,46] may be associated with the prevalence of MDR *E*. *coli* isolates observed in the present study.

A notably high level of antibiotic resistance was observed across all the antibiotics investigated in this study, with a particularly high prevalence in isolates originating from the OD, followed by the HE compared to the KL and SW. This observed pattern of resistance could potentially be attributed to exposure of these bacterial isolates to heavy metals particularly from the OD, which used to be the hub of informal e-waste recycling. Elevated concentrations of heavy metals, including lead (Pb), cadmium (Cd), copper (Cu), nickel (Ni), iron (Fe), and chromium (Cr), exceeding internationally accepted limits [47] have been measured in the water bodies (OD and KL) and soil adjacent to their banks [48–50]. There have been documented instances of bacteria developing resistance to antibiotics as a result of exposure to heavy metals [51,52]. Due to their non-biodegradable nature and tendency to persist in the environment, heavy metals create a persistent reservoir of chemicals, contributing to the co-selection of bacteria resistant to both heavy metals and antibiotics in these environments [53]. This poses a significant public health concern, as individuals residing in close proximity to such environments may be at risk of contracting or becoming carriers of MDR bacteria, presenting challenges in terms of effective treatment. Additionally, drinking water sources and recreational waters [54], farm produce irrigated with contaminated water, and foliage consumed by ruminants could become contaminated and act as reservoirs of ARBs and ARGs. Previous studies have reported that drinking water sources have shown limited effectiveness in the removal of ARGs [55]. The potential consequences of this situation may pose a risk to the integrity of food safety measures and impede progress towards the attainment of the SDGs.

The level of resistance to meropenem, an important carbapenem used for the management of bacterial infections within healthcare facilities in Ghana, is a matter of considerable concern [56]. This is concerning because resistance could potentially be transferred to the clinic, making treatment of infections challenging. This finding aligns with published data from Ghana as reported by Odonkor *et al*., (2022) [54], albeit exhibiting a slightly higher prevalence. Additionally, all *E*. *coli* isolates were completely susceptible to fosfomycin. To date, there is a dearth of reports documenting the presence of fosfomycin resistance in both environmental and clinical sources within the geographical context of Ghana [57,58]. The potential explanation for this phenomenon could be attributed to the high cost of the antibiotic in question and its comparatively reduced usage compared with alternative antibiotics [58]. The results of this study inconvertibly show that there is prevalence of resistance to commonly used antibiotics in patients, thus indicating the potential for this resistance to extend to newer antibiotics such as fosfomycin, which thus far did not show any resistance among all *E*. *coli* isolates. Phenotypically observed isolates categorized as intermediates (neither resistant nor susceptible) can develop resistance over time, either by acquiring ARGs or through chromosomal mutations.

In this study, it was observed that more than 90% of the *E*. *coli* isolates exhibited a minimum MAR index of 0.2 or higher. This finding implies that these isolates likely originated from environments in which they were potentially subjected to the influence of various antibiotics. Therefore, these environmental sources pose a significant risk of the transmission of MDR bacteria among communities residing in close proximity. We recorded MDR isolates that exhibited resistance to a maximum of nine antibiotics in the OD and HE samples, and eight antibiotics in the KL sample. The elevated MAR index may also be attributed to horizontal gene transfer events involving plasmids among various bacterial species within the surrounding ecosystem. The above-mentioned observation aligns with the findings reported by Krumperman (1983) [59] and Grabow and Prozesky (1973) [60], indicating plasmid exchanges between *E*. *coli* and other coliforms in stagnant sections of wastewater systems. An observed MAR index of 0.08 for certain isolates located upstream of the OD and midstream indicates that these isolates may be native to the environment and have not been subjected to extensive antibiotic exposure or acquired plasmids that contribute to MDR. This observation indicates that the pollution of these water bodies may be attributed to external sources, which is indeed the prevailing situation. It is imperative for Ghana to effectively implement policies of recycling of solid waste and the treatment of wastewater before its discharge into the environment. This measure will be crucial for mitigating the escalation of environmental antimicrobial resistance and the subsequent health hazards it poses.

Majority of *E*. *coli* strains isolated from all the sources were primarily associated with phylogroups A and B1, with phylogroup A showing the highest prevalence. A single isolate belonging to phylogroup B2 was isolated from HE. The higher occurrence of phylogroups A and B1, as opposed to B2, aligns with previous research conducted in different environmental settings [61,62]. However, this particular pattern has only been documented in clinical isolates within the Ghanaian context [63]. The elevated occurrence of groups A and B1 can be ascribed to the historically substantial quantities of untreated waste derived from humans and animals that have been discharged into these ecosystems. The co-occurrence of these two groups has frequently been documented in previous studies [61], and their ability to endure environmental stresses in aquatic ecosystems has been reported [64]. *E*. *coli* strains that fall within the A and B1 phylogroups are commensal bacteria found in the gastrointestinal microbiota of vertebrates and aquatic habitats. In contrast, the B2 phylogroup, which consists of pathogenic strains, has been observed in herbivorous and omnivorous mammals [61,65–67].

The *E*. *coli* isolates investigated in this study, classified as part of the B2 group, showed a collective presence of 23 virulence genes. Phylogroup B2 is known to be pathogenic and is responsible for causing extraintestinal *E*. *coli* infections, specifically in humans [61,65,68,69]. Furthermore, it has been demonstrated that B2 strains exhibit a greater ability to survive for longer periods in infants than other strains [70]. In contrast, groups A and B1 contained virulence genes ranging from four to seventeen (4–17). This is consistent with findings documented in previous studies [68,69]. Previous studies have indicated that phylogroups A and B1 exhibit reduced virulence, yet they are still capable of causing intestinal infections in mammals. According to Ochman, Lawrence and Grolsman (2000) [71], it is possible for phylogroups A and B1 to acquire virulence genes, leading to the occurrence of infections. There are reports of the association of phylogroup A with urinary tract infections [72], and A, B1, and B2 [73] with diarrheagenic infections. The presence of these phylogroups possessing the capacity to acquire virulence genes and their circulation within the environment is a matter of significant public health concern because, they can induce severe complications in vulnerable populations, including immunocompromised individuals such as the elderly, children, and convalescents.

Various studies have reported the link between phylogroups and antibiotic resistance [62,65,74]. In this study, phylogroups A and B1 exhibited MAR indices ranging from 0.3 to 0.8, constituting 52% and 44% of the recorded data, respectively. While only one isolate was identified as belonging to phylogroup B2, its MAR index was significantly elevated at 0.6 (1%). This aligns with the findings of previous studies conducted by Mosquito and colleagues [74], which a documented a higher percentage of AR in the B2 group compared to the A and B1 groups. Moreover, several studies [62,65,75] have indicated that the A and B1 groups could be MDR, as observed in this study. This phenomenon may be attributed to HGT [75]. Considering the predisposition of groups, A and B1 to induce gastrointestinal and extra-intestinal infections, the noteworthy prevalence of AR exhibited by these isolates may lead to challenging therapeutic scenarios.

The ESBL-Ec isolates identified from all studied sources, except KL, were found to be present in eight clonal lineages: ST866 (n = 2), ST700, ST390, ST342, ST218, ST202, ST167, and ST21 as well as three novel lineages, namely ST14536, ST14537, and ST14538. These STs are uncommon in other studies from Ghana and Africa. In a different context, the ST21 clone, a subgroup of the Shiga toxin-producing *E*. *coli* clone O26:H11/H-, was identified as the main cause of haemolytic uraemic syndrome (HUS) and bloody diarrhoea [76]. Additionally, an MDR-high-risk clone, ST167, was identified. The presence of ST167 has been reported in poultry and humans in Ghana [44]. Although the two isolates assigned to this ST did not demonstrate any carbapenemase activity, they were identified as ESBLs, suggesting the potential for the development of to carbapenems resistance over time. The potential dissemination of ST21 and ST167 within the environment poses a significant risk to public health, necessitating vigilant monitoring measures to ensure public health protection.

Our study also observed that the ESBL-Ec strains were associated with various β-lactamase genes, including *CTX-M-15*, *TEM-1*, *-35*, *-1B*, *-1C* (ESBLs), *DHA-1* (ampC BLs), and *OXA-1*, *-181* (oxacillinases). This observation is consistent with the findings reported in a study conducted by Delgado-Blas *et al*. (2022) [18]. In their research, the authors detected the presence of comparable genes, such as *CTX-M-15*, *TEM-1*, *-1B*, *-1C*, *DHA-1*, *and OXA-1*, in *Citrobacter spp*. as well as *TEM-1C* in *E*. *coli*, isolated from hospital effluents in the northern region of Ghana. The aforementioned results consistent with similar findings reported in Africa and other regions [14,27,77]. Despite the limited amount of information on the presence of these genes in environmental sources in Ghana, their existence has been recorded in alternative sources such as poultry, animal products, and the surrounding ecosystem [42,44,58,78,79]. The presence of the *CTX-M-15* gene, among the most prevalent CTX-M-type genes, raises a significant public health concern due to its association with the dissemination of MDR bacterial strains and its substantial contribution to nosocomial infections globally [58,80]. Recently, the presence of the OXA-181 gene, which is a variant of the OXA-48 carbapenemase-producing enterobacteriaceae (CPE), was also documented in a clinical setting in Ghana [79,81]. The ubiquity of these β-lactamase genes in the environment implies that their spread may be extensive and can contribute to the rise of AR in healthcare settings. For that reason, its presence in the environment should be closely monitored to control its spread in the community.

The aforementioned *E*. *coli* β-lactamase genes and other ARGs are often disseminated by plasmids. In this study, the Inc plasmid replicon groups were the dominant types with 19 different types of plasmids (including Incl2, IncH1, IncL/M, IncY, IncR, IncQ, IncN and IncF groups). IncF replicons (FIA, FIB, FIC and FII) were the most dominant types (31%), and were found to be resistant to multiple classes of antibiotics, including cephalosporins, sulfamethoxazalole-trimethoprim, carbapenem, and quinolone. These data are consistent with previous reports from environmental sources in Africa and other parts of the world [27,82]. The IncF (IA-II-IB) group of plasmids is frequently found in bacteria isolated from humans, animals, and the environment [83] and is often the carrier of the CTX-M genes in *E*. *coli* [27], as evidenced in this study. These groups of plasmids carry resistance genes for broad-spectrum cephalosporins and other classes of antibiotics including aminoglycosides, trimethoprim, sulphonamides, tetracyclines, and chloramphenicol [84]. Given their presence in Enterobacteriaceae, a bacterial family known for its broad host range, these plasmids can exert a substantial influence on the persistent dissemination of ARGs among humans, animals, and the environment.

This study identified a wide range of VAGs commonly linked to pathogenic *E*. *coli* strains including those that cause diarrheagenic and extraintestinal diseases. The findings of our study are consistent with other studies that have identified similar virulence genes in environmental *E*. *coli* isolates [14,62,85,86]. The phylogroup B1 was observed to carry the highest number of virulent genes, followed by A and B2, respectively. While phylogroups A and B1 are considered commensal strains, B2 is known as a pathogenic strain. Notably phylogroups A and B1 have been associated with urinary tract infections [87], suggesting that commensal strains can become pathogenic by acquiring virulence factors from pathogenic strains in the environment. Virulence genes were associated with specific activities such as colonization (fimH: type 1 fimbriae, csgA: curli fimbriae) in isolates D7_S154 and C7_S158, fitness (fyuA: ferric yersiniabactin uptake) in isolates E2_S39 and D9_S44, toxins (hlyE: alpha haemolysin toxin, astA: enteroaggregative heat-stable toxin, secretory diarrhoea) in isolates F3_S150, D2_S40 and F7_S155. *Escherichia coli* strains in phylogroup B2 are known to be pathogenic, as observed in this study where the isolate originated from a hospital effluent, indicating a human origin. This isolate was found to possess virulence factors associated with serum survival protein (iss), adherence and biofilm formation (afaA-afaD), and the vacuolating autotransporter protein (vat) gene, which codes for a cytotoxin. The presence of both VAGs and ARGs observed across several of the isolates except four isolates (C5, C7, B9 and D1) that showed no ARGs, agrees with other studies where *E*. *coli* was isolated from the environment [88]. This finding contrasts with a study suggesting a trade-off between resistance and virulence of *E*. *coli* [89].

Phylogenomic analyses of isolates sharing the same STs, and phylogenetic groups showed some similarities, suggesting a potential relationship and possible dissemination by both clonal and horizontal spread. This poses a significant risk to public health, as MDR strains may easily propagate across different hosts within a One Health continuum.

Temperature is the primary determinant affecting the survival and growth of *E*. *coli* in the environment [88]. Temperatures recorded for wet and dry seasons ranged between 26.9–31.2°C and there was no observed impact of seasonality on *E*. *coli* strains isolated. Escherichia coli grows at an optimal temperature of 37–44°C. This implies that in tropical regions characterized by consistently high temperatures, *E*. *coli* has the potential to persist in contaminated environments throughout the year. Overall, understanding the complex interplay between seasonality and environmental *E*. *coli* is crucial for assessing water quality and potential health risks, implementing effective management strategies to control *E*. *coli* levels, and predicting changes in *E*. *coli* concentrations under future climate scenarios. The research on seasonality and environmental *E*. *coli* is ongoing, and more data is needed to fully understand the intricate interactions across diverse ecosystems. By continuing to investigate these dynamics, we can better protect the health of our environment and communities.

## Conclusions

The findings of our study highlight the intricate and varied genomic attributes exhibited by MDR *E*. *coli* isolated from urban polluted environmental sources. The presence of strains exhibiting ESBL activity and carrying the blaCTX-M-15 gene has implications for clinical practice and a matter of significant public health concern. This gene has been linked to the extensive spread of MDR bacteria on a global scale. It is noteworthy that the presence of ST21 has not been documented in clinical isolates within the Ghanaian context. Furthermore, the detection of IncF plasmids and MGEs, which play a crucial role in promoting the dissemination of these ARGs, was also noted. The findings indicate that public health is at risk and requires urgent attention. To address this issue, it is imperative to develop and implement effective strategies pertaining to waste management. By doing so, we can effectively mitigate the discharge of ARB into the environment and, curtail the dissemination of ARGs. The results of this study provide evidence for the necessity of conducting AMR surveillance studies that encompass the One Health approach. Usage of high-throughput technologies such as WGS is crucial to gain genomic insights on the AMR status in Ghana, with the goal of protecting public health. The present study contributes to the expansion of our understanding of the resistance mechanisms and pathogenic attributes of *E*. *coli*, which facilitate its proliferation and dissemination within environmental niches.

## Policy implications

The implications of this study are profound for both policy formulation and practical interventions. The evident insufficiency of proper waste treatment infrastructure in Ghana emerges as a key determinant of AMR proliferation, demanding immediate attention and resolution. Addressing the issue of inadequate waste management is intricate, involving various sectors and requiring the concerted efforts of multiple stakeholders, prominently the government. A critical component of this effort is the implementation of policies and actions, with a particular focus on providing robust Water, Sanitation, and Hygiene (WASH) facilities, especially in urban slums.

Recognizing the pivotal role of the media in disseminating information, it becomes essential for effective public education aimed at fostering behavioral change regarding waste management practices. Concurrently, the establishment of comprehensive public health surveillance is imperative to monitor AMR. Environmental officers hold the responsibility of ensuring compliance with waste management regulations, adding another layer to the multifaceted solution required.

Moreover, adopting a One Health approach is indispensable for the surveillance of antibiotic-resistant bacteria and genes. This approach facilitates evidence-based decision-making, particularly crucial in resource-poor settings like Ghana. By integrating insights from human, animal, and environmental health, a holistic understanding of the extent of the problem can be achieved, enabling more effective strategies to combat antimicrobial resistance.

## Supporting information

S1 Fig Frequency of *Escherichia coli* isolates showing number of antibiotic resistances. (TIF)

S2 Fig Frequency of *Escherichia coli* isolates from sampling sites showing number of antibiotic resistances. (TIF)

S3 Fig Percentage of *Escherichia coli* isolates from the sampling sites and their phenotypic resistance to 12 antibiotics. Odaw River (OD), Korle Lagoon, sewage (SW) and hospitals effluents (HE), AMP—Ampicillin, CXM—Cefuroxime, CTX—Cefotaxime, CAZ—Ceftazidime, CRO—Ceftriaxone, CIP—Ciprofloxacin, AZM—Azithromycin, AK—Amikacin, MEM—Meropenem, C—Chloramphenicol, AK—Amikacin, SXT-Sulfamethoxazole-Trimethoprim.
(TIF)

S4 Fig Resistance patterns of *Escherichia coli* isolates. (TIF)

S5 Fig Distribution of sequence types (STs) among 25 *Escherichia coli* isolates. (TIF)

S6 Fig Distribution of Phylogroups among 25 *Escherichia coli* isolates. (TIF)

S7 Fig Distribution of phylogroups among *Escherichia coli* isolates from sample sources. (TIF)

S8 Fig Distribution of acquired resistance genes amongst *Escherichia coli* isolates. (TIF)

S9 Fig Distribution of virulence genes across *E*. *coli* isolates. (TIF)

S10 Fig Distribution of plasmids amongst *Escherichia coli* isolates. (TIF)

S1 Table Source and co-ordinates of sampling locations. (DOCX)

S2A Table Genomic characteristics of E. coli isolates downloaded from the BVBR 3.30.19a. (DOCX)

S2B Table Metadata of *E*. *coli* isolates downloaded from the BVBR 3.30.19a. (XLSX)

S3 Table Antibiotic resistance and susceptibility values of *Escherichia coli* isolates. (DOCX)

S4 Table Source and resistant profiles of *Escherichia coli* isolates. (DOCX)

S5 Table Genomic characteristics of *E*. *coli* isolate. (DOCX)

## Abbreviations

ABR: antibiotic-resistant bacteria
AMR: Antimicrobial Resistance
AR: Antibiotic resistance
AST: antibiotic susceptibility testing
CGE: Center for Genomic Epidemiology
*E. coli*: Escherichia coli
ESBL: Extended spectrum β-lactamase
e-waste: electronic waste
ExPEC: extraintestinal pathogenic *E*. *coli*
HE: hospital effluent
HGT: horizontal gene transfer
KL: Korle Lagoon
LMICs: low-and middle income countries
MALDITOF MS: matrix-assisted laser desorption/ionization-time of flight mass spectrometry
MAR: Multiple Antibiotic Resistance
MDR: multidrug resistant
MGEs: mobile genetic elements
MLST: multilocus sequence typing
OD: Odaw River
PCR: polymerase chain reaction
qPCR: quantitative PCR
SE: sewage effluents
SNPs: single-nucleotide polymorphisms
ST: sequence types
UPEC: uropathogenic *E*. *coli*
VAGs: virulence-associated genes
WGS: whole genome sequencing
WHO: World Health Organization
WWTP: wastewater treatment plant
