# Supplementary figures and images for: Genomic analysis of multidrug-resistant Escherichia coli from Urban Environmental water sources in Accra, Ghana, Provides Insights into public health implications
Source: PLoS One. 2024 May 24;19(5):e0301531. doi: 10.1371/journal.pone.0301531 (PMC11125565; doi:10.1371/journal.pone.0301531)

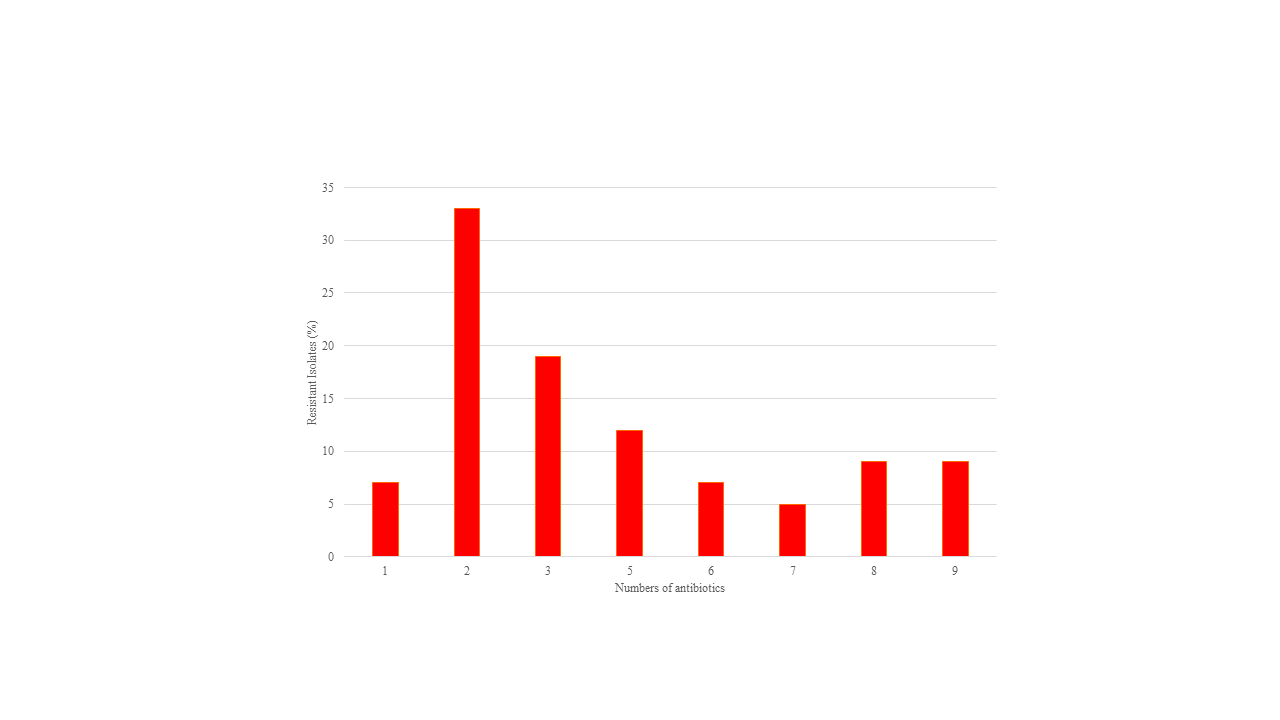

Supplement: S1 Fig — (TIF) [file pone.0301531.s001.tif]

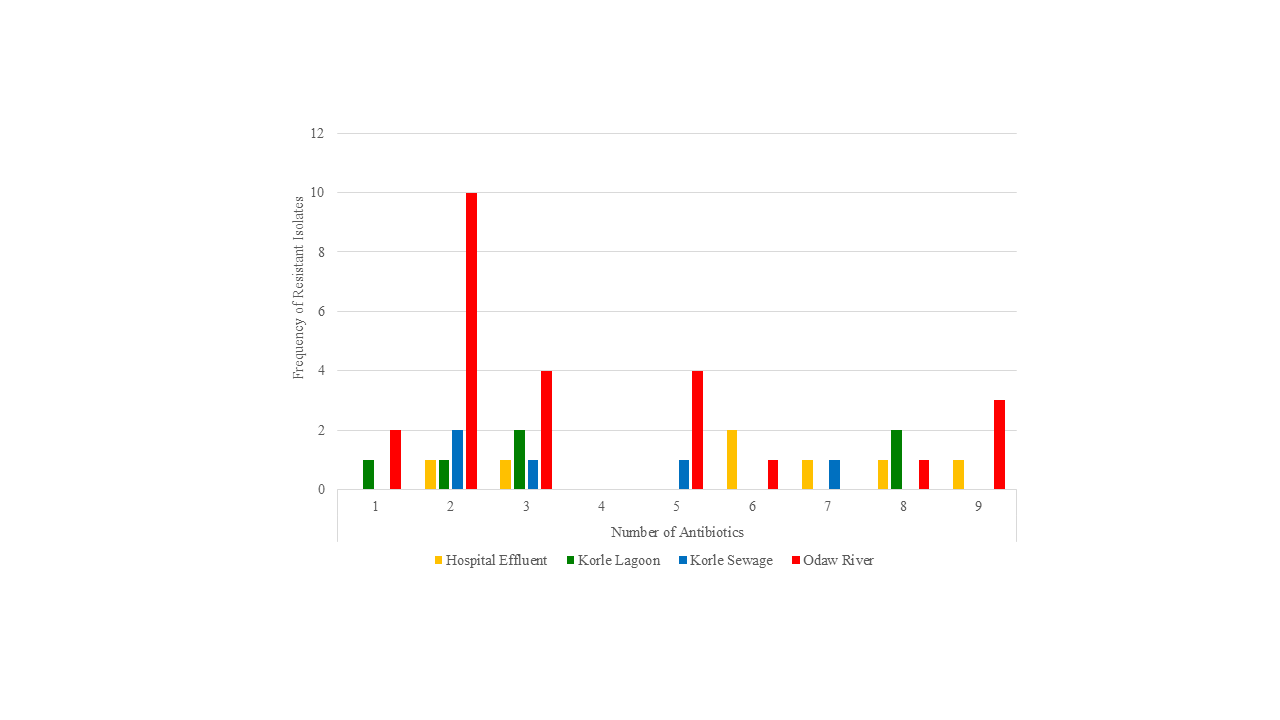

Supplement: S2 Fig — (TIF) [file pone.0301531.s002.tif]

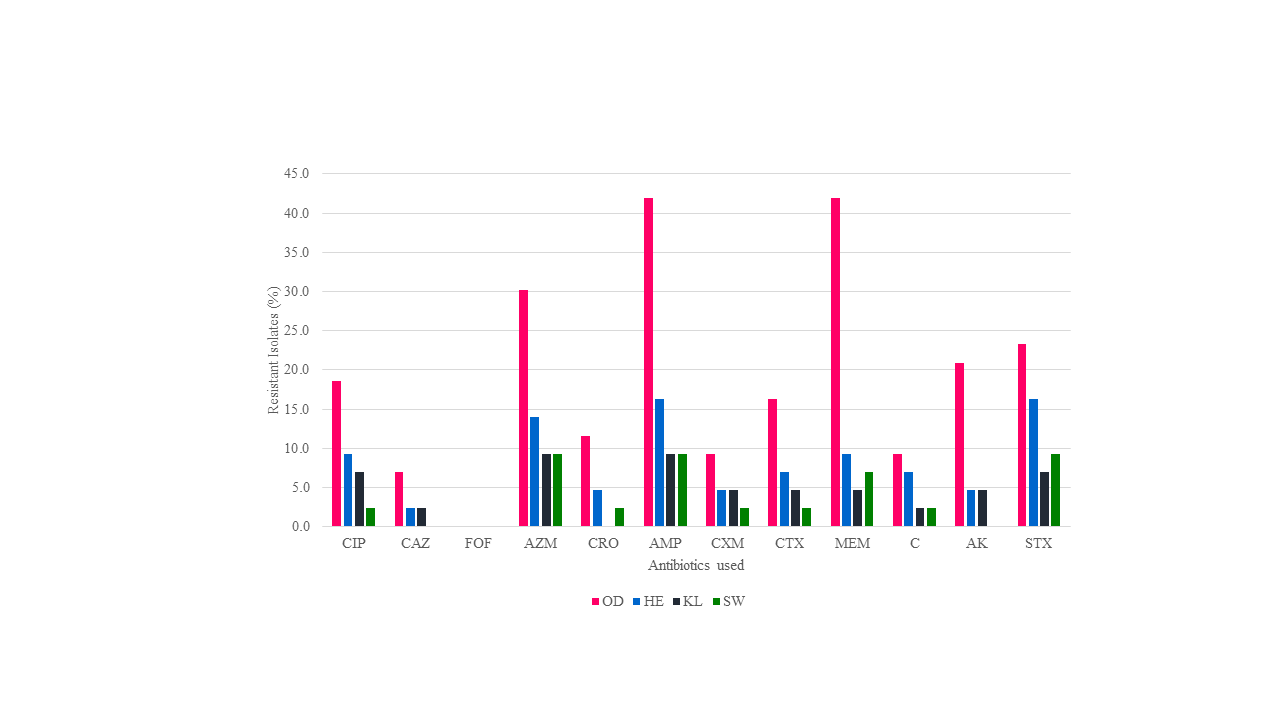

Supplement: S3 Fig — Odaw River (OD), Korle Lagoon, sewage (SW) and hospitals effluents (HE), AMP—Ampicillin, CXM—Cefuroxime, CTX—Cefotaxime, CAZ—Ceftazidime, CRO—Ceftriaxone, CIP—Ciprofloxacin, AZM—Azithromycin, AK—Amikacin, MEM—Meropenem, C—Chloramphenicol, AK—Amikacin, SXT-Sulfamethoxazole-Trimethoprim. (TIF) [file pone.0301531.s003.tif]

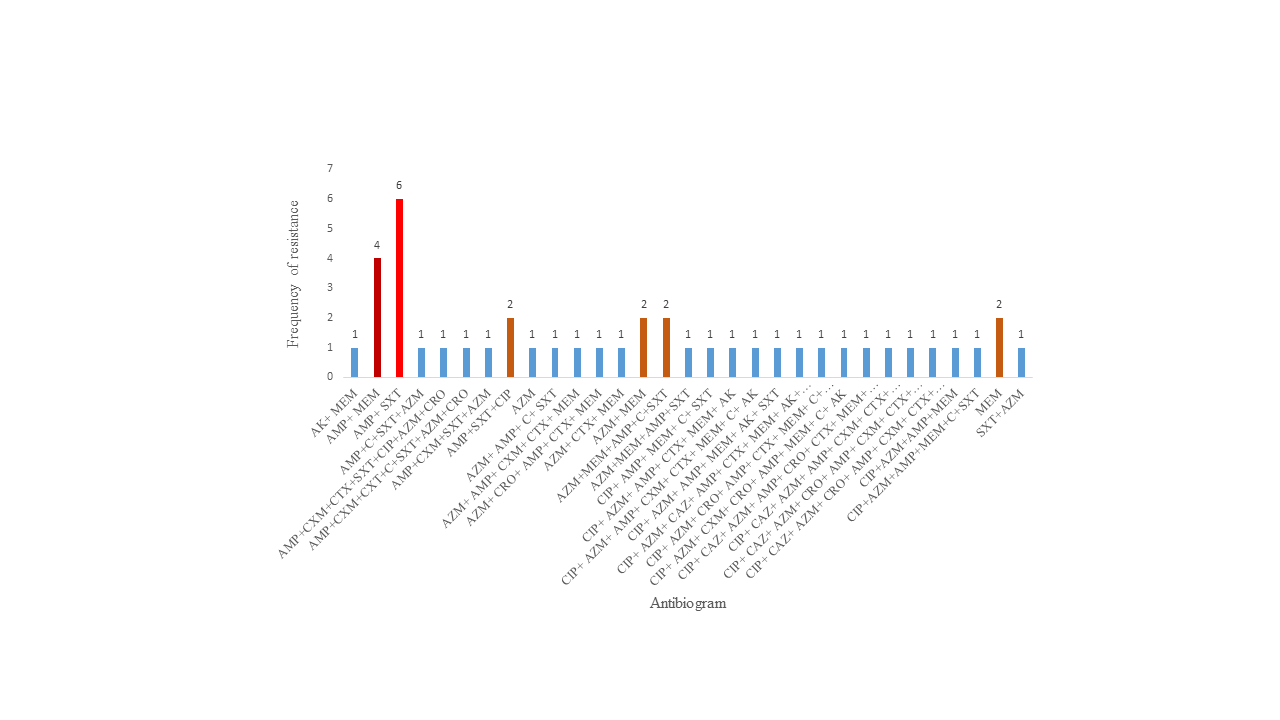

Supplement: S4 Fig — (TIF) [file pone.0301531.s004.tif]

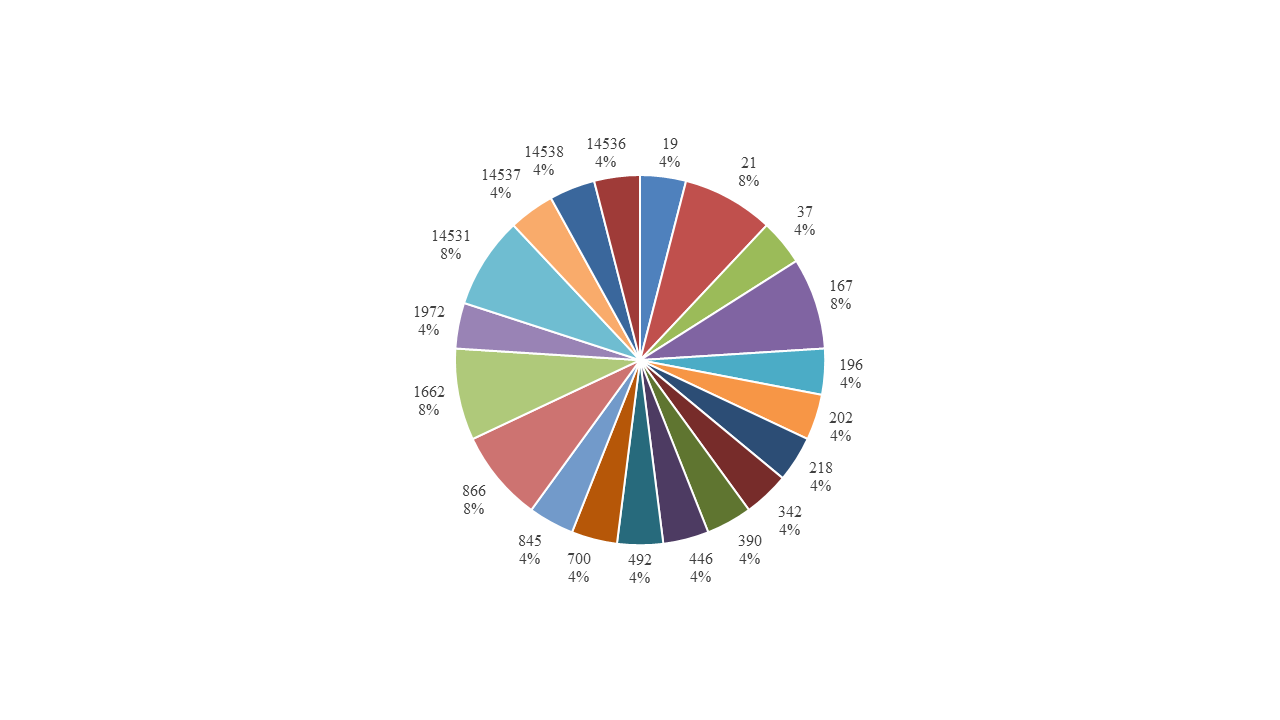

Supplement: S5 Fig — (TIF) [file pone.0301531.s005.tif]

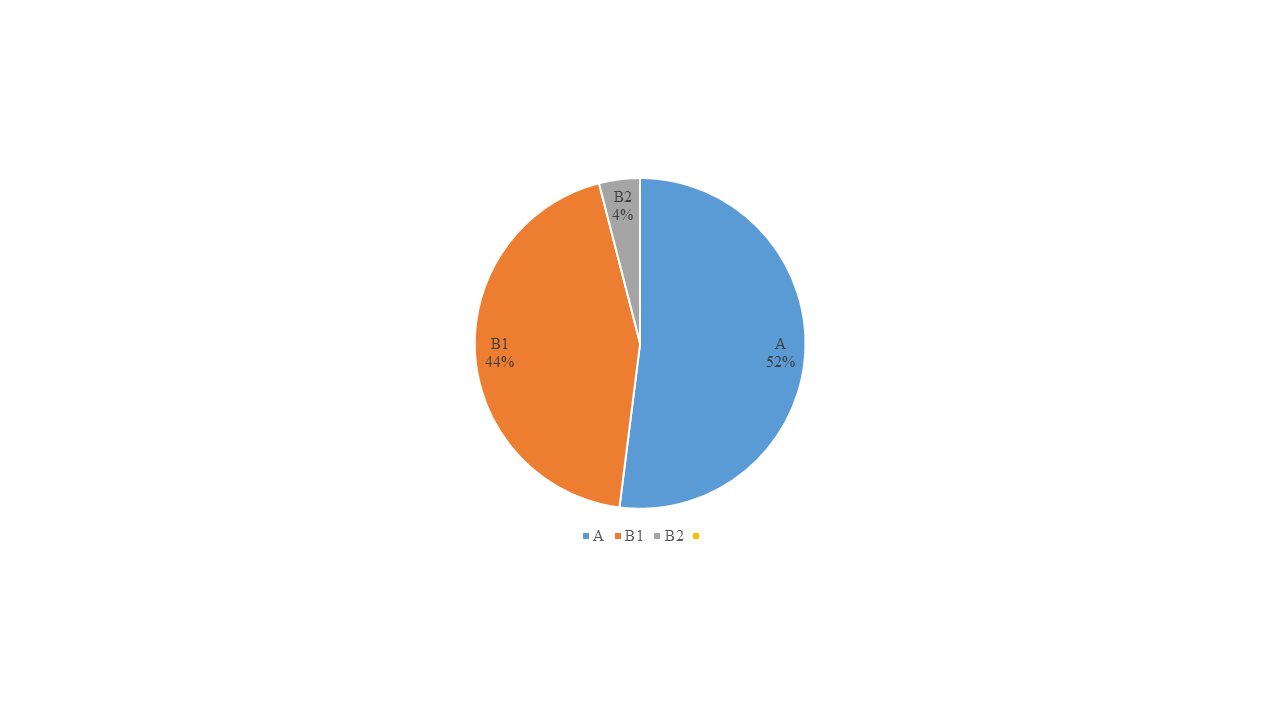

Supplement: S6 Fig — (TIF) [file pone.0301531.s006.tif]

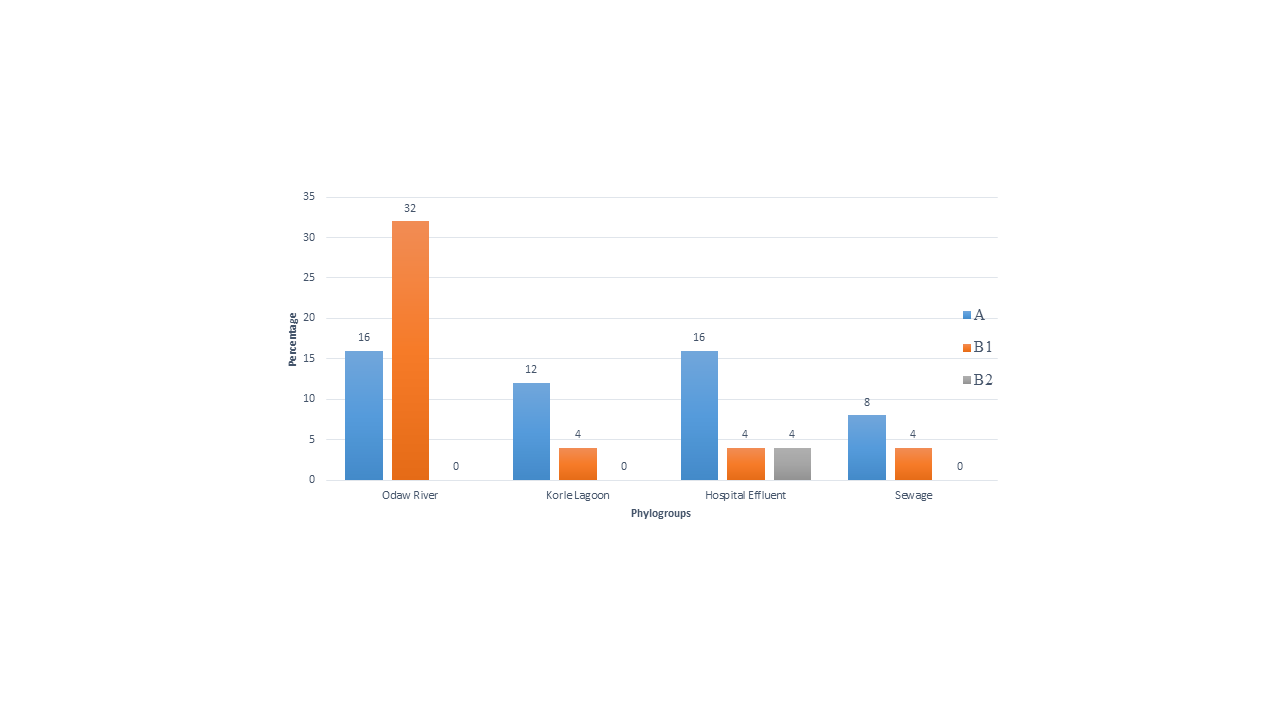

Supplement: S7 Fig — (TIF) [file pone.0301531.s007.tif]

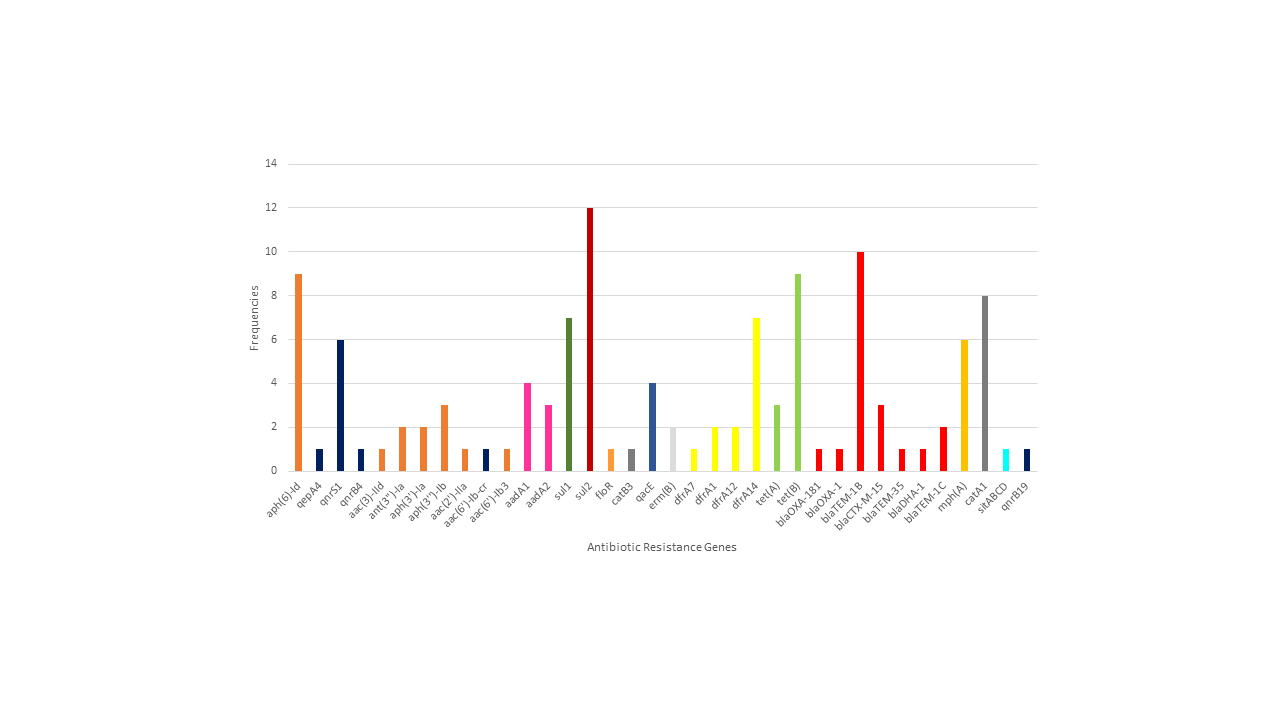

Supplement: S8 Fig — (TIF) [file pone.0301531.s008.tif]

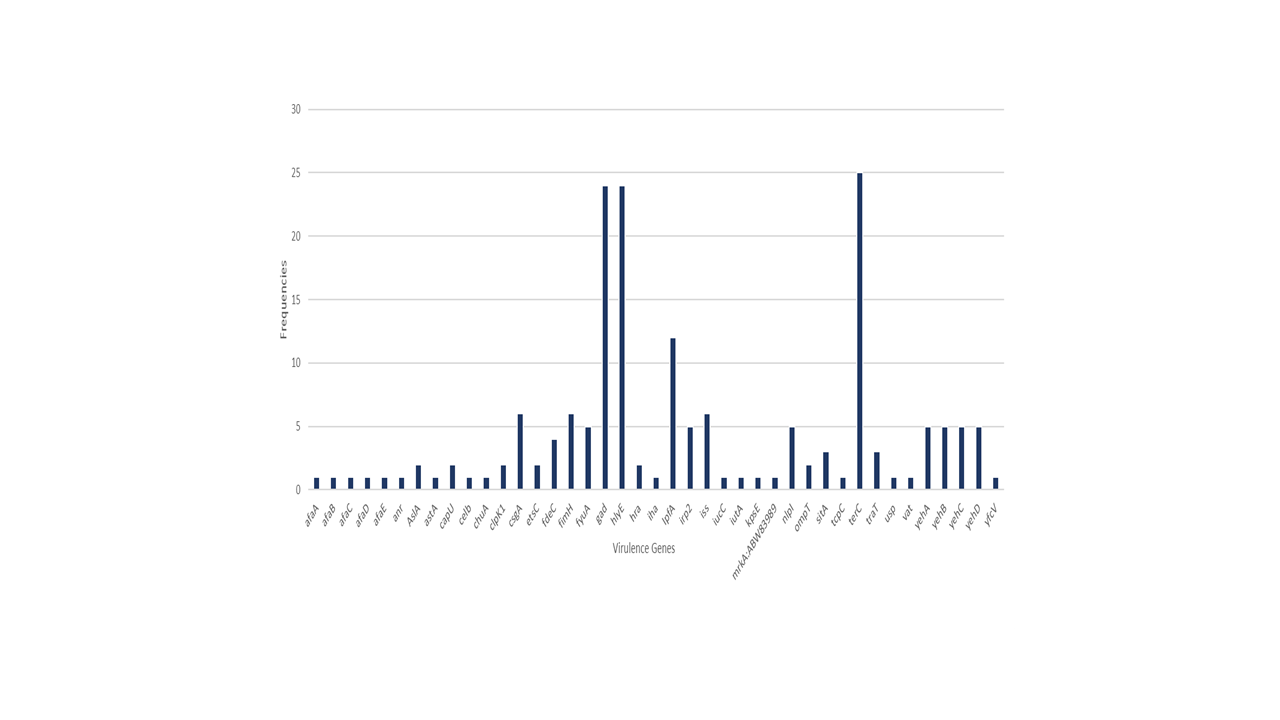

Supplement: S9 Fig — (TIF) [file pone.0301531.s009.tif]

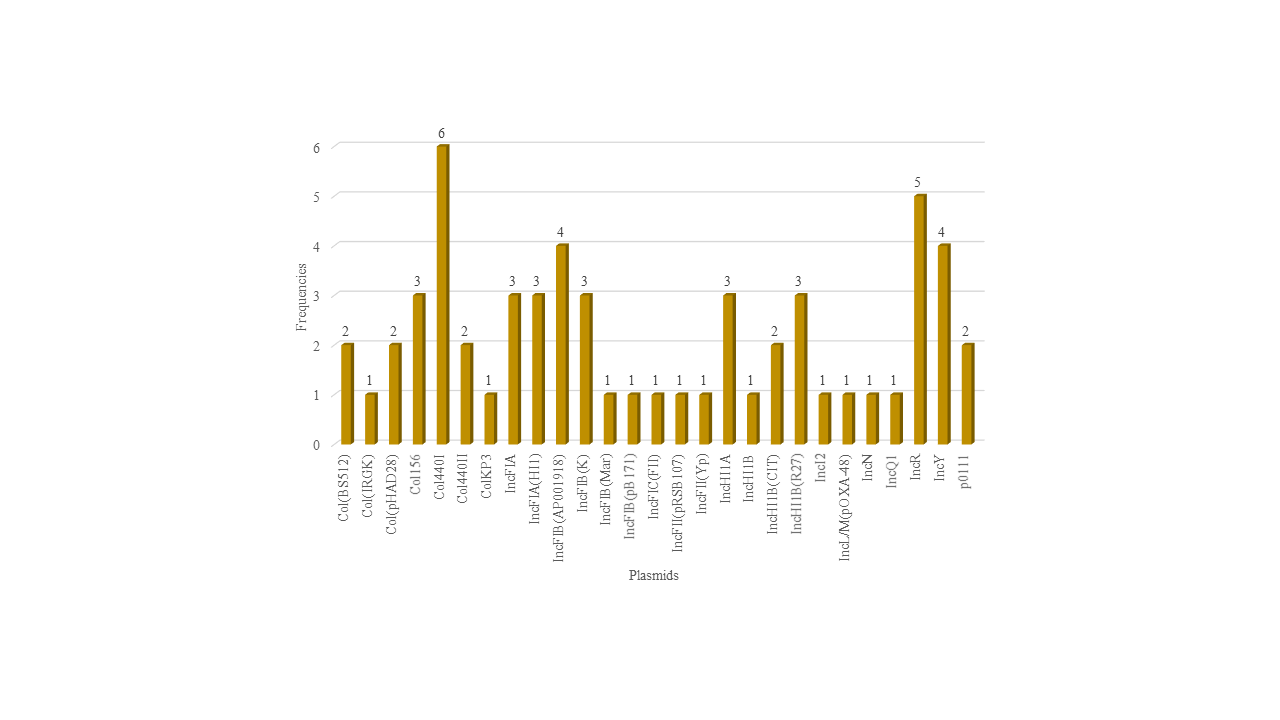

Supplement: S10 Fig — (TIF) [file pone.0301531.s010.tif]
