# Supplementary material for: Genomic analysis of multidrug-resistant Escherichia coli from Urban Environmental water sources in Accra, Ghana, Provides Insights into public health implications
Source: PLoS One. 2024 May 24;19(5):e0301531. doi: 10.1371/journal.pone.0301531 (PMC11125565; doi:10.1371/journal.pone.0301531)
Supplement: S1 Table — (DOCX) [file pone.0301531.s011.docx]

S1 Table. Source and co-ordinates of sampling locations

| Site | Site code | Source | Year | Latitude | Longitude |
| --- | --- | --- | --- | --- | --- |
| Odaw downstream | ODDS1 | water | 2021 | 5.542528 | -0.224639 |
| Odaw upstream | ODUS1 | water | 2021 | 5.556417 | -0.221639 |
| Korle sewage | KLSW1 | water | 2021 | 5.536722 | -0.219139 |
| Odaw downstream | ODDS2 | water | 2021 | 5.542528 | -0.224583 |
| Odaw upstream | ODUS2 | water | 2021 | 5.55325 | -0.223889 |
| Hospital effluent | LH | water | 2021 | 5.649910 | -0.178100 |
| Korle Lagoon MS | KLMS2 | water | 2021 | 5.538028 | -0.220694 |
| Hospital effluent | 37 MH | water | 2021 | 5.591260 | -0.188290 |
| Hospital effluent | KBTH | water | 2021 | 5.542028 | -0.227685 |
| Korle sewage | KLSW2 | water | 2021 | 5.534667 | -0.218833 |
| Korle sewage | KLSW2 | water | 2021 | 5.534667 | -0.218833 |
| Odaw midstream | ODMS3 | water | 2021 | 5.549972 | -0.22525 |
| Hospital effluent | 37 MH (1.2) | sediment | 2021 | 5.591260 | -0.188290 |
| Hospital effluent | 37 MH (1.2) | sediment | 2021 | 5.591260 | -0.188290 |
| Hospital effluent | 37 MH (W) | water | 2021 | 5.591260 | -0.188290 |
| Korle Lagoon DS | KLDS1 | water | 2021 | 5.531472 | -0.221528 |
| Odaw midstream | ODMS3 | water | 2021 | 5.549972 | -0.22525 |
| Odaw midstream | ODMS3 | sediment | 2021 | 5.549972 | -0.22525 |
| Odaw downstream | ODDS1 | water | 2021 | 5.542528 | -0.224639 |
| Odaw midstream | ODMS3.2 | water | 2021 | 5.549972 | -0.22525 |
| Hospital effluent | 37 MH (1.2) | sediment | 2021 | 5.591260 | -0.188290 |
| Odaw downstream | ODDS1 | water | 2021 | 5.542528 | -0.224639 |
| Korle Lagoon DS | KLDS1 | water | 2021 | 5.531472 | -0.221528 |
| Korle sewage | KLSW2 | water | 2021 | 5.534667 | -0.218833 |
| Odaw midstream | ODMS3.2 | water | 2021 | 5.549972 | -0.22525 |
| Odaw midstream | ODMS3.2 | water | 2021 | 5.549972 | -0.22525 |
| Korle Lagoon DS | KLDS2 | water | 2021 | 5.531472 | -0.221528 |
| Odaw midstream | ODMS3 | water | 2021 | 5.549972 | -0.22525 |
| Korle Lagoon DS | KLDS1 | water | 2021 | 5.531472 | -0.221528 |
| Korle sewage | KLSWIW | water | 2021 | 5.536722 | -0.219139 |
| Korle Lagoon DS | KLDS1W | water | 2021 | 5.531472 | -0.221528 |
| Odaw upstream | ODUSIW | water | 2021 | 5.556417 | -0.221639 |
| Odaw upstream | ODUS1W | sediment | 2021 | 5.556417 | -0.221639 |
| Odaw upstream | ODUS1W | sediment | 2021 | 5.556417 | -0.221639 |
| Odaw upstream | ODUS1W | sediment | 2021 | 5.556417 | -0.221639 |
| Odaw upstream | ODUS1W | sediment | 2021 | 5.556417 | -0.221639 |
| Odaw upstream | ODGUS2W* | sediment | 2021 | 5.556528 | -0.221611 |
| Odaw upstream | ODUS2W | sediment | 2021 | 5.556528 | -0.221611 |
| Odaw upstream | ODGUS2W* | sediment | 2021 | 5.556528 | -0.221611 |
| Odaw upstream | ODGUS2W* | sediment | 2021 | 5.556528 | -0.221611 |
| Odaw upstream | ODGUS2W* | sediment | 2021 | 5.556528 | -0.221611 |
| Odaw upstream | ODGUS1W | sediment | 2021 | 5.55644444 | 0.22163889 |
| Odaw upstream | ODUS2W | sediment | 2021 | 5.556528 | -0.221611 |
