# Supplementary material for: Genomic analysis of multidrug-resistant Escherichia coli from Urban Environmental water sources in Accra, Ghana, Provides Insights into public health implications
Source: PLoS One. 2024 May 24;19(5):e0301531. doi: 10.1371/journal.pone.0301531 (PMC11125565; doi:10.1371/journal.pone.0301531)
Supplement: S2A Table — (DOCX) [file pone.0301531.s012.docx]

S2 Table. Genomic characteristics of *E. coli* isolates downloaded from the BVBR 3.30.19a

(<https://www.bv-brc.org/view/Taxonomy/561#view_tab=genomes>)

| Isolates | Source | STs | Phylogroup | Beta-lactam |
| --- | --- | --- | --- | --- |
| EC0001_Hu_18_Gh.fasta | Human | ST2006 | B1 | blaCTX-M-15 |
| EC0002_Hu_18_Gh.fasta | Human | ST2006 | B1 | blaCTX-M-15 |
| EC0003_Hu_18_Gh.fasta | Human | ST2006 | B1 | blaCTX-M-15 |
| EC0004_Hu_18_Gh.fasta | Human | ST2006 | B1 | blaCTX-M-15 |
| EC0005_Hu_18_Gh.fasta | Human | ST2006 | B1 | blaCTX-M-15 |
| EC0006_Hu_18_Gh.fasta | Human | ST2006 | B1 | blaCTX-M-15 |
| EC0007_Hu_18_Gh.fasta | Human | ST10 | A | blaCTX-M-15,blaOXA-1 |
| EC0008_Hu_18_Gh.fasta | Human | ST131 | B2 | blaCTX-M-15,blaOXA-1 |
| EC0009_Hu_18_Gh.fasta | Human | ST156 | B1 | blaCTX-M-15,blaTEM-1B |
| EC001_Pig_20_Gh.fasta | Pig | ST167 | A | blaCTX-M-15 |
| EC0010_Hu_18_Gh.fasta | Human | ST156 | B1 | blaCTX-M-15,blaTEM-1B |
| EC0011_Hu_18_Gh.fasta | Human | ST69 | D | blaOXA-1,blaTEM-169,blaTEM-33 |
| EC0012_Hu_18_Gh.fasta | Human | ST131 | B2 | blaCTX-M-15,blaOXA-1 |
| EC0013_Hu_18_Gh.fasta | Human | ST131 | B2 | blaCTX-M-15,blaOXA-1 |
| EC0014_Hu_18_Gh.fasta | Human | ST131 | B2 | blaCTX-M-15,blaOXA-1 |
| EC0015_Hu_18_Gh.fasta | Human | ST46 | A | blaTEM-1B |
| EC0016_Hu_18_Gh.fasta | Human | ST131 | B2 | blaCTX-M |
| EC0017_Hu_18_Gh.fasta | Human | ST131 | B2 | blaCTX-M-15,blaOXA-1 |
| EC0018_Hu_18_Gh.fasta | Human | ST12 | B2 | blaTEM-1B |
| EC0019_Hu_18_Gh.fasta | Human | ST449 | D | blaOXA-1 |
| EC002_Pig_20_Gh.fasta | Pig | ST58 | B1 | blaCTX-M-15,blaTEM-1B |
| EC0020_Hu_18_Gh.fasta | Human | ST5747 | B1 | Unknown |
| EC0021_Hu_18_Gh.fasta | Human | ST38 | D | blaTEM-1C |
| EC004_Pig_20_Gh.fasta | Pig | ST9967 | B1 | blaCTX-M-15,blaTEM-1B |
| EC005_Pig_20_Gh.fasta | Pig | ST58 | B1 | blaCTX-M-15,blaCTX-M-180 |
| EC006_Pig_20_Gh.fasta | Pig | ST9138 | B1 | Unknown |
| EC007_Pig_20_Gh.fasta | Pig | ST167 | A | blaCTX-M-15 |
| EC008_Pig_20_Gh.fasta | Pig | ST206 | A | blaCTX-M-15,blaTEM-1B |
| EC009_Pig_20_Gh.fasta | Pig | ST196 | B1 | blaCTX-M-15 |
| EC010_Pig_20_Gh.fasta | Pig | ST410 | C | blaCTX-M-15,blaOXA-1,blaTEM-1B |
| EC010_Pou_20_Gh.fasta | Poultry | ST1706 | B1 | blaCTX-M-15 |
| EC011_Pig_20_Gh.fasta | Pig | ST4450 | A | blaCTX-M-15 |
| EC012_Pig_20_Gh.fasta | Pig | ST226 | A | blaCTX-M-15 |
| EC013_Pig_20_Gh.fasta | Pig | ST10 | A | blaCTX-M-15,blaTEM-1B |
| EC014_Pou_20_Gh.fasta | Poultry | ST1140 | E | blaCTX-M-15 |
| EC014A_Pig_20_Gh.fasta | Pig | ST12818 | A | blaCTX-M-15 |
| EC015_Pig_20_Gh.fasta | Pig | ST10 | A | blaCTX-M-15 |
| EC016_Pig_20_Gh.fasta | Pig | 38* | D | blaCTX-M-15 |
| EC017_Pig_20_Gh.fasta | Pig | ST38 | D | blaCTX-M-15,blaTEM-1B |
| EC018_Pig_20_Gh.fasta | Pig | ST165 | A | blaCTX-M-15 |
| EC019_Pig_20_Gh.fasta | Pig | ST3580 | B1 | blaCTX-M-15 |
| EC020_Pig_20_Gh.fasta | Pig | ST9967 | B1 | blaCTX-M-15,blaTEM-1B |
| EC021_Pig_20_Gh.fasta | Pig | ST1727 | B1 | blaCTX-M-15 |
| EC022_Pig_20_Gh.fasta | Pig | ST206 | B1 | blaCTX-M-15,blaTEM-1B |
| EC023_Pig_20_Gh.fasta | Pig | ST196 | B1 | blaCTX-M-15,blaTEM-1B |
| EC028_Pou_20_Gh.fasta | Poultry | ST5044 | B1 | blaCTX-M-55 |
| EC030_Pou_20_Gh.fasta | Poultry | ST3018 | E | Unknown |
| P72_Hu_21_Gh.fasta | Human | ST167 | A | blaCTX-M-27 |
