# Supplementary material for: Genomic analysis of multidrug-resistant Escherichia coli from Urban Environmental water sources in Accra, Ghana, Provides Insights into public health implications
Source: PLoS One. 2024 May 24;19(5):e0301531. doi: 10.1371/journal.pone.0301531 (PMC11125565; doi:10.1371/journal.pone.0301531)
Supplement: S3 Table — (DOCX) [file pone.0301531.s014.docx]

S3 Table. Antibiotic resistance and susceptibility values of *E. coli* isolates (N = 57).

| Antimicrobial agents | Antibiotic  Classes | Breakpoint for resistance (μg/mL) | Resistance (%) | Susceptible (%) | Intermediates (%) |
| --- | --- | --- | --- | --- | --- |
| Ciprofloxacin | Quinolone | (5 µg) S≥31/I 20-30/R≤20 | 17(29.8) | 6(10.5) | 34(59.6) |
| Ceftazidime | Cephalosporin | (30 µg) S≥21/I 18-20/R≤17 | 5(8.8) | 47(82.5) | 5(8.8) |
| Fosfomycin | Phosphonic acid  derivatives | (200 µg) S≥16/I 13-15/R≤12 | 0(0) | 100(100) | 0(0) |
| Azithromycin | Macrolide | (15 µg) S≥13/R≤12 | 26(45.6) | 31(54.4) | 0(0) |
| Ceftriaxone | Cephalosporin | (30 µg) S≥23/I 20-22/R≤19 | 8(14.0) | 42(73.7) | 7(12.3) |
| Ampicillin | Penicillin | (10 µg) S≥17/I 14-16/R≤13 | 36(63.2) | 17(29.8) | 4(7.0) |
| Cefuroxime | Cephalosporin | (30 µg) S≥23/I 15-22/R≤14 | 10(17) | 4(7) | 45(76) |
| Cefotaxime | Cephalosporin | (30 µg) S≥26/I 23-25/R≤22 | 14(24) | 35(59) | 10(17) |
| Meropenem | Carbapenem | (10 µg) S≥23/I 20-22/R≤19 | 27(47.4) | 30(52.6) | 0(0) |
| Chloramphenicol | Amphenicol | (30 µg) S≥18/I 13-17/R≤12 | 10(17.5) | 39(68.4) | 8(14.0) |
| Amikacin | Aminoglycoside | (30 µg) S≥17/I 15-16/R≤14 | 12(21.1) | 33(57.9) | 12(21.1) |
| Sulfamethoxazole-Trimethoprim | Sulfonamide | (1.25/23.75 µg) S≥16/I 11-15/R≤10 | 24(42.1) | 31(54.4) | 2(3.5) |

N - Number of *E. coli* isolates, R – Resistant, S – Susceptible
