# Supplementary material for: Genomic analysis of multidrug-resistant Escherichia coli from Urban Environmental water sources in Accra, Ghana, Provides Insights into public health implications
Source: PLoS One. 2024 May 24;19(5):e0301531. doi: 10.1371/journal.pone.0301531 (PMC11125565; doi:10.1371/journal.pone.0301531)
Supplement: S4 Table — (DOCX) [file pone.0301531.s015.docx]

S4 Table. Source and resistant profiles of *Escherichia coli* isolates

| Site | Profile | CIP  n(%) | CAZ  n(%) | FOS  n(%) | AZM  n(%) | CRO  n(%) | AMP  n(%) | CXM  n(%) | CTX  n(%) | MEM  n(%) | C  n(%) | AK  n(%) | SXT  n(%) |
| --- | --- | --- | --- | --- | --- | --- | --- | --- | --- | --- | --- | --- | --- |
| Hospital effluent | Resistant | 4(9.3) | 1(2.3) | 0(0.0) | 6(14.0) | 2(4.7) | 7(16.3) | 2(4.7) | 3(7.0) | 4(9.3) | 3(7.0) | 2(4.7) | 7(16.3) |
|  | Susceptible | 0(0.0) | 5(11.6) | 7(16.3) | 1(2.3) | 5(11.6) | 0(0.0) | 2(4.7) | 3(7.0) | 3(7.0) | 3(7.0) | 2(4.7) | 0(0.0) |
|  | Intermediate | 3(7) | 1(2.3) | 0(0.0) | 0(0.0) | 0(0.0) | 0(0.0) | 3(7.0) | 1(2.3) | 0(0.0) | 1(2.3) | 3(4.7) | 0(0.0) |
| Korle Lagoon | Resistant | 3(7) | 1(2.3) | 0(0.0) | 4(9.3) | 0(0.0) | 4(9.3) | 2(4.7) | 2(4.7) | 2(4.7) | 1(2.3) | 2(4.7) | 3(7.0) |
|  | Susceptible | 1(2.3) | 5(11.6) | 6(14.0) | 2(4.7) | 3(7.0) | 2(4.7) | 0(0.0) | 3(7.0) | 4(9.3) | 5(11.6) | 3(7.0) | 2(4.7) |
|  | Intermediate | 2(4.7) | 0(0.0) | 0(0.0) | 0(0.0) | 3(7.0) | 0(0.0) | 4(9.3) | 1(2.3) | 0(0.0) | 0(0.0) | 1(2.3) | 1(2.3) |
| Sewage | Resistant | 1(2.3) | 0(0.0) | 0(0.0) | 4(9.3) | 1(2.3) | 4(9.3) | 1(2.3) | 1(2.3) | 3(7.0) | 1(2.3) | 0(0.0) | 4(4.0) |
|  | Susceptible | 1(2.3) | 3(7.0) | 5(11.6) | 1(2.3) | 4(9.3) | 1(2.3) | 0(0.0) | 4(9.3) | 2(4.7) | 3(7.0) | 3(7.0) | 1(2.3) |
|  | Intermediate | 3(7.0) | 2(4.7) | 0(0.0) | 0(0.0) | 0(0.0) | 0(0.0) | 4(9.3) | 0(0.0) | 0(0.0) | 1(2.3) | 2(4.7) | 0(0.0) |
| Odaw River | Resistant | 8(18.6) | 3(7.0) | 0(0.0) | 13(30.2) | 5(11.6) | 18(41.9) | 4(9.3) | 7(16.3) | 18(41.9) | 4(9.3) | 9(20.9) | 10(23.3) |
|  | Susceptible | 1(2.3) | 19(44.2) | 21(48.8) | 12(27.9) | 16(37.2) | 3(7.0) | 2(4.7) | 12(27.9) | 6(14.0) | 15(34.9) | 6(14.0) | 13(30.2) |
|  | Intermediate | 16(37.2) | 3(7.0) | 4(9.3) | 0(0.0) | 4(9.3) | 4(9.3) | 19(44.2) | 6(14.0) | 1(2.3) | 6(14.0) | 10(23.3) | 2(4.7) |

AMP – Ampicillin, CXM – Cefuroxime, CTX – Cefotaxime, CAZ - Ceftazidime, CRO – Ceftriaxone, CIP – Ciprofloxacin, AZM – Azithromycin, AK – Amikacin, MEM – Meropenem, C – Chloramphenicol, AK – Amikacin, SXT-Sulfamethoxazole-Trimethoprim
