# Supplementary material for: Genomic analysis of multidrug-resistant Escherichia coli from Urban Environmental water sources in Accra, Ghana, Provides Insights into public health implications
Source: PLoS One. 2024 May 24;19(5):e0301531. doi: 10.1371/journal.pone.0301531 (PMC11125565; doi:10.1371/journal.pone.0301531)
Supplement: S5 Table — (DOCX) [file pone.0301531.s016.docx]

S5 Table. Genomic characteristics of *E. coli* isolate

| Genome Name | Genome Length | No. Contigs | Smallest Contig | Largest Contig | Average Contig Length | N50 | L50 | non-ATCG | GC Content |
| --- | --- | --- | --- | --- | --- | --- | --- | --- | --- |
| E6_S42 | 5049158 | 151 | 204 | 320679 | 33438 | 143874 | 12 | 0 | 50.5 |
| E12_S41 | 4514053 | 100 | 201 | 353729 | 45140 | 128422 | 12 | 0 | 50.7 |
| B11_S156 | 4833397 | 107 | 218 | 643514 | 45171 | 193301 | 7 | 0 | 50.7 |
| B9_S166 | 4656909 | 62 | 225 | 581032 | 75111 | 356403 | 5 | 0 | 50.8 |
| F9_S161 | 4691281 | 138 | 201 | 254395 | 33994 | 106190 | 16 | 0 | 50.9 |
| F7_S155 | 5277882 | 156 | 200 | 272781 | 33832 | 135095 | 15 | 0 | 50.6 |
| C1_S167 | 4702985 | 82 | 207 | 403306 | 57353 | 235883 | 8 | 0 | 50.8 |
| B12_S163 | 4705607 | 70 | 207 | 417198 | 67222 | 207632 | 9 | 0 | 50.8 |
| D9_S44 | 4803461 | 156 | 204 | 370635 | 30791 | 135551 | 12 | 0 | 50.7 |
| F5_S151 | 4864030 | 132 | 202 | 317906 | 36848 | 129632 | 13 | 0 | 50.7 |
| D7_S154 | 5151195 | 122 | 200 | 489774 | 42222 | 160106 | 11 | 0 | 50.6 |
| F3_S150 | 4593259 | 99 | 204 | 358964 | 46396 | 119195 | 13 | 0 | 50.8 |
| D3_S43 | 4646039 | 128 | 202 | 385172 | 36297 | 121176 | 13 | 0 | 50.8 |
| D1_S162 | 4665009 | 71 | 204 | 522164 | 65704 | 234044 | 6 | 0 | 50.8 |
| B7_S168 | 4659669 | 66 | 230 | 640568 | 70601 | 288841 | 6 | 0 | 50.6 |
| D5_S152 | 4934702 | 133 | 205 | 247653 | 37103 | 129887 | 14 | 0 | 50.4 |
| F1_S159 | 4771265 | 90 | 200 | 817257 | 53014 | 209287 | 7 | 0 | 50.6 |
| D2_S40_L001 | 4896383 | 147 | 201 | 254436 | 33308 | 125565 | 15 | 0 | 50.8 |
| F8_S165 | 5000556 | 176 | 201 | 224589 | 28412 | 73630 | 19 | 0 | 50.6 |
| C7_S158 | 4666555 | 80 | 204 | 522104 | 58331 | 235816 | 6 | 0 | 50.8 |
| E2_S39_L001 | 5007895 | 144 | 212 | 366753 | 34777 | 247654 | 8 | 0 | 50.6 |
| D12_S164 | 4651917 | 103 | 224 | 428757 | 45164 | 129502 | 11 | 0 | 50.8 |
| D8_S149 | 4751386 | 224 | 200 | 232097 | 21211 | 73523 | 20 | 0 | 50.9 |
| C5_S153 | 4618343 | 131 | 201 | 299919 | 35254 | 118788 | 14 | 0 | 50.7 |
| F4_S157 | 4519772 | 132 | 215 | 260617 | 34240 | 72849 | 20 | 0 | 50.9 |
